# Risk Factors for Infections, Antibiotic Therapy, and Its Impact on Cancer Therapy Outcomes for Patients with Solid Tumors

**DOI:** 10.3390/life11121387

**Published:** 2021-12-11

**Authors:** Ondřej Kubeček, Pavla Paterová, Martina Novosadová

**Affiliations:** 1 Department of Oncology and Radiotherapy, Faculty of Medicine and University Hospital in Hradec Králové, Charles University, Sokolská 581, 50005 Hradec Králové, Czech Republic; ondrej.kubecek@fnhk.cz; 2 Department of Clinical Microbiology, Faculty of Medicine and University Hospital in Hradec Králové, Charles University, Sokolská 581, 50005 Hradec Králové, Czech Republic; 3 Department of Clinical Pharmacy, Hospital Pharmacy, University Hospital in Hradec Králové, Sokolská 581, 50005 Hradec Králové, Czech Republic; martina.novosadova@fnhk.cz

**Keywords:** infection, risk factors, antibiotic therapy, cancer, solid tumors, targeted therapy, immunotherapy

## Abstract

Infections represent a significant cause of morbidity and mortality in cancer patients. Multiple factors related to the patient, tumor, and cancer therapy can affect the risk of infection in patients with solid tumors. A thorough understanding of such factors can aid in the identification of patients with substantial risk of infection, allowing medical practitioners to tailor therapy and apply prophylactic measures to avoid serious complications. The use of novel treatment modalities, including targeted therapy and immunotherapy, brings diagnostic and therapeutic challenges into the management of infections in cancer patients. A growing body of evidence suggests that antibiotic therapy can modulate both toxicity and antitumor response induced by chemotherapy, radiotherapy, and especially immunotherapy. This article provides a comprehensive review of potential risk factors for infections and therapeutic approaches for the most prevalent infections in patients with solid tumors, and discusses the potential effect of antibiotic therapy on toxicity and efficacy of cancer therapy.

## 1. Introduction

Patients with solid tumors have an increased risk of infectious complications [1]. There are several factors contributing to the susceptibility of cancer patients to infections. The tumor itself affects the host immune system by inducing a catabolic state and malnutrition, which leads to immunosuppression [2]. Tumor growth can cause obstruction of tubular organs and disruption of anatomical barriers, leading to penetration of bacteria into the bloodstream [2,3]. Radiation therapy, targeted agents, and cytotoxic drugs used in cancer therapy may further increase the risk of infections by inducing neutropenia, mucositis, and skin toxicity [3].

The incidence of sepsis is 16.4 cases per 1000 cancer patients per year, with in-hospital mortality of 37.8% in severe cases [4]. Bloodstream infections are significantly more common in patients with hematologic malignancies than in those with solid tumors. Interestingly, while there is a significant downward trend in bloodstream infection in hematologic patients over time, the incidence remains stable in patients with solid tumors [5]. Infections are one of the leading causes of mortality in cancer patients, and the rate of fatal infections is approximately three times higher than that of the general population [6]. This underscores the importance of appropriate management of infections in cancer patients.

## 2. Immunosuppression in Cancer Patients

Patients with solid tumors are generally considered to be immunocompromised, with the level of immunosuppression depending on the tumor, patient characteristics, and therapy regimen used [7]. There are a number of differences between patients with solid tumors and those with hematologic malignancies. First, the neoplastic process of those with solid tumors does not involve the effectors of the host immune system. Second, the therapy of solid tumors usually does not lead to prolonged neutropenia [3]. Consequently, the incidence of infections in patients with solid tumors is significantly lower compared to hematologic patients [5,8,9]. Prophylactic use of antibiotics is, therefore, rarely used for patients with solid tumors. A possible exception is the use of trimethoprim/sulfamethoxazole for the prevention of *Pneumocystis jirovecii* pneumonia in patients receiving temozolomide together with radiation therapy, and moderate-to-high-dose corticosteroid therapy (prednisone equivalents ≥ 20 mg for ≥4 weeks) [7].

## 3. Immunosuppression Induced by Chemotherapy

The immunosuppressive activity of cytotoxic drugs is usually expressed as their potential to induce febrile neutropenia (FN; defined as oral temperature of >38.3 °C or two consecutive readings of >38.0 °C for two hours together with an absolute neutrophil count [ANC] of <0.5 × 10^9^/L, or expected to decline below 0.5 × 10^9^/L) [10]. Chemotherapy regimens are usually divided into three risk groups according to the likelihood of FN: high risk (>20%), intermediate risk (10–20%), and low risk (<10%). Common chemotherapy regimens for the therapy of solid tumors with a significant risk of FN are listed in Table 1. Patients treated with high-risk regimens benefit from the primary prophylactic use of granulocyte colony-stimulating factors (G-CSF) [7,10,11]. The indication of G-CSF use in the intermediate-risk group is based on the presence of additional risk factors, including age > 65 years, advanced disease, history of FN, no antibiotic prophylaxis, poor performance status, patient’s comorbidities, open wounds, previous chemotherapy or radiation therapy, bone marrow involvement with tumor, and poor nutritional status [11,12].

## 4. Immunosuppression Induced by Targeted Therapy

Besides cytostatic therapy, some targeted agents used in the therapy of solid tumors show immunosuppressive activity. Inhibitors of the mammalian target of rapamycin (mTOR inhibitors—everolimus, temsirolimus) are known to induce immunosuppression by altering the balance between effector T cells and regulatory T cells (Tregs), and inhibiting NK cells, B cells, antigen presenting cells, neutrophils, and mast cells [47]. Consequently, patients treated with mTOR inhibitors have an increased risk of all-grade (RR = 2.00; 95% CI, 1.76–2.28, P < 0.001) and high-grade (RR = 2.60; 95% CI, 1.54–4.41, P < 0.001) infections [48]. Most infections affect the respiratory tract (61.7%), followed by genitourinary tract (29.4%) and skin/soft tissue (4.2%) infections [48]. In addition, patients treated with mTOR inhibitors can develop non-infectious pneumonitis, which may cause diagnostic problems [49]. Currently, there are no specific recommendations for antimicrobial prophylaxis in patients treated with mTOR inhibitors, although a high level of alertness is required [50].

Other targeted agents, such as BRAF kinase inhibitors (dabrafenib, encorafenib, vemurafenib) and multi-target protein kinase inhibitors (sorafenib), do not show a direct immunosuppressive effect, but the therapy may be associated with neutropenia and/or lymphocytopenia [7]. Similarly, therapy with cyclin-dependent kinase 4/6 (CDK 4/6) inhibitors used in the therapy of breast cancer (palbociclib and ribociclib) is associated with a high incidence of neutropenia [51]. However, despite this relatively high incidence, few patients develop FN, and infectious complications are rare [51]. This can be attributed to the fact that CDK 4/6 inhibitor-induced bone marrow suppression occurs through cell-cycle arrest with no apoptosis and is therefore reversible upon therapy withdrawal [52]. This is in contrast to chemotherapy, which can induce apoptosis of hematopoietic precursor and progenitor cells. Furthermore, mucositis and skin toxicity, which are considered risk factors for infections in chemotherapy-related neutropenia, are rare in CDK 4/6 inhibitor therapy [51]. Leucopenia is rare in another CDK 4/6 inhibitor, abemaciclib, which is explained by it being a more potent inhibitor of CDK4 than CDK6 [53].

Immune checkpoint inhibitors (ICIs), including anti-CTLA-4, anti-PD-1, and anti-PD-L1 monoclonal antibodies, do not confer a higher risk for infectious complications. However, they can induce immune-related adverse events (irAEs), necessitating the use of corticosteroids and other immunosuppressive drugs [50]. Adequate prophylaxis should be used, depending on the level of induced immunosuppression and the drugs used [7]. One of the most common irAEs—immune-mediated colitis—can lead to disruption of the mucosal barrier, resulting in bacteremia and intestinal perforation with peritonitis [54].

## 5. Risk Scores for Febrile Neutropenia

Considering the complexity of risk factors for FN, there has been an effort to develop risk scores capable of predicting the risk for individual patients. Several studies developed risk scores for FN [55,56,57,58,59], some of which have been prospectively validated [56,58]. The overview of these studies, including independent risk factors for FN, is shown in Table 2. Aagard et al. introduced an internally validated risk score for FN during the first cycle of chemotherapy (the FENCE Score) [57]. Recently, an update providing cycle-specific risk for FN (during cycle 2–6) in solid tumors has been published [59]. Such risk scores could be used to predict the risk of FN in a particular patient and initiate preventive measures, including the use of G-CSF in high-risk patients, although external validation in large prospective trials is required [59].

## 6. Risk Factors for Infections in Patients with Solid Tumors

**Figure 1:**
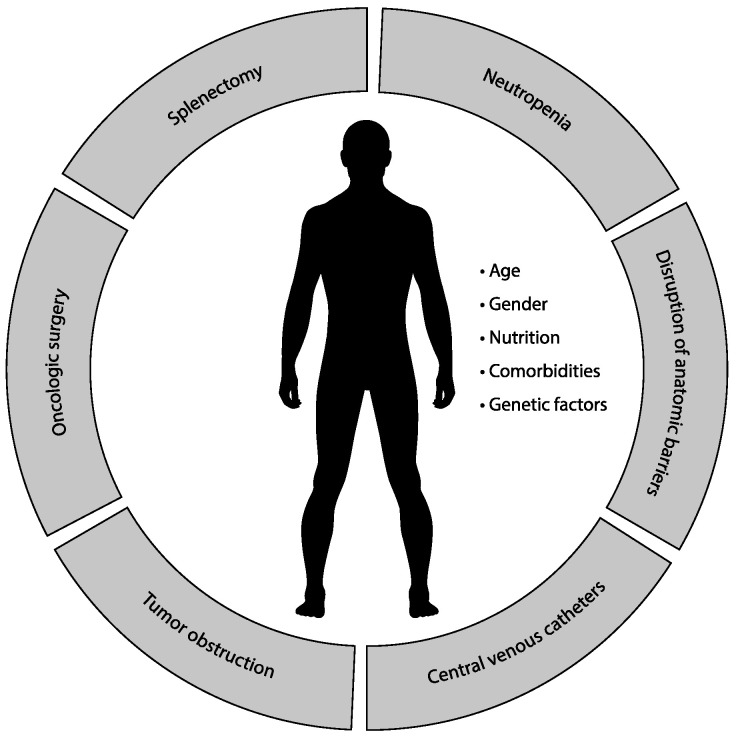
Factors affecting risk of infection.

Patients with solid tumors are a heterogeneous group regarding the risk of infection complications [1]. The National Comprehensive Cancer Network (NCCN) guidelines determine three risk categories based on the overall risk of developing an infection (low-, intermediate-, and high-risk categories) [7]. Most patients with solid tumors belong to the low-risk (standard chemotherapy regimens with anticipated neutropenia <7 days) and intermediate-risk categories (anticipated neutropenia 7–10 days) [7]. Besides the depth and duration of immunosuppression, the presence of specific risk factors must be considered. The most common are depicted in Figure 1. Usually, multiple risk factors are combined in one patient [3].

### 6.1. Neutropenia

Severe neutropenia (grade 4) is defined as the ANC of <500 cells/mm^3^ according to the Common Terminology Criteria for Adverse Events (CTCAE) [60]. A drop of ANC below this threshold is associated with an increased risk of infections [61]. The incidence and severity of infections are inversely related to ANC, with the highest risk when ANC drops below 100 cells/mm^3^. Duration of neutropenia is another important risk factor. Chemotherapy regimens resulting in neutropenia lasting >10 days are considered high risk [7]. However, most regimens used in the therapy of solid tumors result in neutropenia lasting <7 days and are therefore considered low risk [7].

### 6.2. Disruption of Anatomic Barriers

Mucositis is another important factor increasing the risk of bloodstream infections. Mucosal barriers in the gastrointestinal, urogenital, and respiratory tract constitute the first line of host defense against various pathogens [7]. The damage of mucosal barriers opens the colonizing pathogens’ gateway to the bloodstream. Coexisting neutropenia allows for the rapid development of severe infections, even in the case of a low bacterial load [62]. Chemotherapy-induced mucositis is associated with an increased risk of infections caused by viridans group streptococci, gram-negative rods, and *Candida* spp. [7]. Although the risk and grade of mucositis is higher in hematologic patients receiving high-dose chemotherapy regimens with autologous hematopoietic stem cell transplantation, some of the cytotoxic drugs used in the therapy of solid tumors can induce mucositis. These include 5-fluorouracil, capecitabine, cyclophosphamide, ifosfamide, cisplatin, carboplatin, docetaxel, paclitaxel, and vinorelbine [3]. A high risk of oral mucositis is associated with some chemotherapy regimens, including DCF (86%), FOLFIRI (80%), CAF (79%), AC (71%), and FOLFOX (60%) [63]. Mucosal toxicity is also frequent in some targeted therapies, including bevacizumab, erlotinib, gefitinib, lapatinib, sorafenib, and sunitinib [64]. Oral mucositis is a typical adverse event in patients treated with mTOR inhibitors, affecting as much as 73% of patients [65]. Additionally, mucositis is a common adverse event in radiation therapy [66].

### 6.3. Central Venous Catheters

Central venous catheters (CVCs) are widely used in cancer patients and offer benefits to those who receive chemotherapy. However, the presence of CVCs is considered a risk factor for infections in cancer patients and may affect the etiology of bacteremia [62]. The incidence of CVC-associated bloodstream infections in cancer patients is estimated to be 0.5–10 per 1000 CVC days, with mortality ranging from 12% to 40% [67].

There are two main types of indwelling CVCs used for chemotherapy administration—centrally inserted totally implanted vascular access ports (PORTs) and peripherally inserted central catheters (PICCs) [68]. PICCs are an alternative to traditional PORTs, and their use has increased owing to their lower cost and ease of insertion and removal [68]. Several studies have compared these CVCs in terms of infection complications, with conflicting results [68,69,70]. A recent meta-analysis found a significantly higher risk of infectious complications in the PICC groups (RR 3.43; 95% CI 2.58–4.56; P < 0.05) [69]. Both the local infections of punctures and catheter-related infections were more frequent in patients with PICCs [69]. In addition, infection complications are more frequent in multi-lumen CVCs than in single-lumen CVCs [71].

Most CVC infections originate from the skin flora (65%), catheter or catheter joints (30%), or other pathways (5%) [72]. The most commonly detected pathogens causing CVC-related infections in cancer patients are coagulase-negative staphylococci, followed by other gram-positive bacteria, including *Staphylococcus aureus,* enterococci, and streptococci [67,73]. As in other bloodstream infections in cancer patients, a shift toward gram-negative flora (including *Escherichia coli*, *Pseudomonas aeruginosa*, and *Klebsiella* spp.) has been noted in CVC-related infections [74,75]. Therefore, a broad-spectrum empirical therapy covering gram-negative pathogens should be considered in CVC-related bloodstream infections [74].

### 6.4. Tumor Obstruction

Direct expansion of the tumor may cause obstruction of tubular organs [3]. Bronchial obstruction caused by local growth of bronchogenic carcinomas and metastatic tumors may cause post-obstructive pneumonia and occasionally be the first manifestation of the disease [76]. The post-obstructive component can be found in ~45–55% of patients with pulmonary neoplasms who develop pneumonia [77]. Bronchial obstruction develops more commonly in tumors arising centrally, such as small cell lung cancer (SCLC) and squamous cell carcinoma (SCC) [78]. Pathogens causing post-obstructive pneumonia include gram-positive organisms (*St. aureus* [incl. MRSA], viridans group streptococci, beta-hemolytic streptococci [groups A, B, C, F, and G]), gram-negative organisms (*E. coli*, *Klebsiella* spp., other Enterobacteriaceae, *Ps. aeruginosa*, *Stenotrophomonas maltophilia*, *Acinetobacter* spp.), anaerobes (*Peptococcus* spp., *Peptostreptococcus* spp., *Fusobacterium nucleatum*, *Bacteroides* spp.), and fungi (*Candida* spp.) [79]. Despite therapy with broad-spectrum antibiotics, responses tend to be low, and persistent or recurrent infections are common. Moreover, ~10–15% of patients develop severe complications, including lung abscess, empyema, hemorrhage, and fistula formation [77].

Obstructive uropathy is a frequent complication of advanced solid tumors, especially prostate, retroperitoneal, and pelvic tumors [80]. It is usually managed by placing a ureteral stent or permanent nephrostomy tube (PNT) [81]. However, the presence of PNT in cancer patients is associated with a high risk of PNT-associated pyelonephritis (with a rate of 19%) [82]. Patients with a history of previous urinary tract infection (UTI) and neutropenia are at higher risk [82]. Many pathogens are capable of forming biofilms on indwelling implants, including *E. coli*, *Enterococcus faecalis*, *Ps. aeruginosa*, *Proteus mirabilis*, *St. aureus*, and *Candida* spp. [82]. Eradication of these microorganisms is challenging, and long-term suppressive antibiotic therapy may be required in patients with recurrent urosepsis [3]. Nevertheless, prophylactic antibiotic therapy does not seem to prevent the development of pyelonephritis and asymptomatic bacteriuria in cancer patients with PNT [82]. In addition to complicated UTIs, patients with prostatic carcinoma may develop prostatitis and prostatic abscesses [3].

Malignant biliary obstruction (MBO) is caused by pancreatic adenocarcinoma, cholangiocarcinoma, ampullary/duodenal adenocarcinoma, gallbladder carcinoma, lymphoma, and compressive metastatic proximal lymph nodes [83]. Pancreatic adenocarcinoma is the most common cause of MBO, and as many as 70–90% of pancreatic cancer patients develop jaundice during the course of their disease [84]. MBO frequently results in ascending cholangitis, which may be the initial manifestation of underlying malignancy [3]. In cases of persistent obstruction, hepatic abscesses may develop [85]. The etiology is usually polymicrobial, with enteric gram-negative rods, *Enterococcus* spp., and anaerobes being most common [86]. Management of MBO includes percutaneous transhepatic biliary drainage [87] and endoscopic retrograde cholangiopancreatography (ERCP) with the placement of plastic or self-expandable metallic stents (SEMS), which is considered the current mainstay of treatment [84,88]. In patients with resectable disease, up-front surgery without a stent placement is an option and does not increase the risk of complications compared to preoperative biliary drainage [84].

Additionally, large tumors can overgrow the capacity of their blood supply and become necrotic, forming the seeds of infection. Direct invasion of colorectal cancer through the mucosa may lead to abscess formation and sepsis by enteric bacteria [7].

### 6.5. Oncologic Surgery

Patients who undergo extensive tumor resection are at increased risk for postoperative nosocomial infection, especially in tumors involving the respiratory and gastrointestinal tract [1]. In a large retrospective study, the rate of serious postoperative infections was 9.4% and led to a nearly 12-fold increase in the odds of in-hospital mortality [89]. Surgical procedures associated with the highest risk of serious infections were esophagectomy (25%), gastrectomy (19%), pancreas resection (17%), and lung resection (10%). The incidence of infections is significantly lower in high-volume centers than in low-volume hospitals [89]. Besides the type of surgery, the risk of infection may be related to the tumor burden, preoperative performance status, and previous therapy [7]. However, it seems that surgical and intensive care unit-related factors play a more significant role than previous oncologic therapy [62].

### 6.6. Splenectomy and Function Asplenia

The number of indications for splenectomy in cancer patients has declined over the years. In the case of solid tumors, splenectomy is traditionally performed in gastric cancer to dissect the splenic hilar lymph nodes, although it seems to have no benefit in tumors located at lesser curvature [90]. Splenectomy may also be performed in cases of oligometastatic disease from other sites, especially ovarian cancer [91]. Additionally, intraoperative splenic injury during abdominal surgery may result in splenectomy [92]. Besides surgical splenectomy resulting in asplenia, radiotherapy and some pathologic conditions (including graft versus host disease following allogeneic hematopoietic stem cell transplantation) lead to a decreased function of the spleen—hyposplenism (function asplenia) [7].

The spleen is a lymphoid organ that plays an important role in regulating immune homeostasis through both innate and adaptive immunity. The function of the spleen is crucial in the elimination of encapsulated bacteria [93]. Asplenic patients are therefore at risk of sepsis caused by encapsulated bacteria—most commonly *Streptococcus pneumoniae* (50–70%), but also *Haemophilus influenzae* and *Neisseria meningitidis* (15–25% each) [94]. Other pathogens causing serious infections in asplenic patients include *Capnocytophaga canimorsus* after animal bites, *Bordetella holmesii*, *Ehrlichia* spp., and intraerythrocytic parasites such as *Babesia* spp. after tick bites [94].

### 6.7. Patient-Related Factors

#### 6.7.1. Age

Age is an important factor regarding the risk for infections. Age-related changes in the immune system, referred to as immunosenescence, play a major role in increased susceptibility to infections in elderly patients [95]. Other risk factors for infections frequently present in elderly patients include malnutrition, comorbidities, age-related organ changes, functional dysfunction (i.e., impairments in the performance of activities of daily living), polypharmacy, and social factors [96,97]. Furthermore, age is associated with an increased risk of FN [98], owing to reduced bone marrow reserves and reduced renal and hepatic functions [99].

#### 6.7.2. Gender

Men are at increased risk for most infections. The explanation for this observation is rather complex and involves both biological and social factors [100]. Sexual steroid hormones play an important role in susceptibility to infections through differential modulation of pro-inflammatory and anti-inflammatory cytokine expression, toll-like receptor expression, antibody production, metabolism, growth, and virulence of pathogenic bacteria [101]. Estrogens can enhance both cell-mediated and humoral immune responses, while progesterone and testosterone have anti-inflammatory effects and suppress innate immune responses [100,101,102]. Additionally, it seems that genetic factors related to sex chromosomes (X and Y) may play a part [103], and differences in occupational activities and lifestyle result in different exposures to pathogens [100].

In contrast to the overall lower risk of infections in the female population, UTIs and genital tract infections are more common in women owing to anatomic and physiological differences [104]. Available data suggest that female gender is a risk factor for the development of FN [98,105]. One possible explanation is that female patients are more frequently treated with breast cancer chemotherapy regimens, which confer a higher risk for FN (up to 23% in standard chemotherapy and 98% in high-dose chemotherapy regimens) [106]. Gender-related differences in pharmacokinetics and pharmacodynamics of anticancer drugs provide another rationale [107]. A recent study has found that polymorphisms of genes involved in drug metabolism are distributed unevenly in women and men, and that these polymorphisms have different impacts on adverse event occurrence (including FN) between genders [108].

#### 6.7.3. Nutrition

Malnutrition affects as many as 75% of cancer patients, with the highest prevalence in those with tumors of the gastrointestinal tract [109]. The association between malnutrition and infections is well-established and can be explained by impaired cell-mediated immunity, phagocyte function, cytokine production, and complement system function [110]. Therefore, improvement of the patient’s nutritional status is of great importance to reduce the risk of infections and improve survival [111].

Obesity is associated with an increased risk of infections when compared to normal-weight subjects [112,113]. The highest increase was observed for skin infections in both genders and for gastrointestinal tract infections, UTIs, and sepsis in obese women [113]. The underlying mechanisms involve altered adipokine signaling (e.g., leptin, adiponectin), immune system dysregulation, impaired chemotaxis, and metabolic changes [112,114].

#### 6.7.4. Comorbidities

Many internal diseases are associated with an increased risk of infection. Patients with type 1 and 2 diabetes are at increased risk of all infections, particularly bone and joint infections, sepsis, and cellulitis [115]. Chronic kidney disease is associated with an increased risk of infections, especially in patients undergoing hemodialysis [116,117]. Patients with chronic obstructive pulmonary disease (COPD) are more likely to develop respiratory infections, but the incidence of infections outside the respiratory tract does not seem to be affected [118]. An increased risk of infections is observed in patients with rheumatological disorders owing to both altered function of the immune system and immunosuppressive therapy [119]. Opportunistic infections, including *P. jirovecii,* are particularly common [120]. Other comorbidities with increased risk of infection include chronic heart failure [121] and cirrhosis [122].

#### 6.7.5. Genetic Factors

Defects in innate and adaptive immunity are naturally associated with an increased risk of infections [123]. Single nucleotide polymorphisms (SNPs) in genes involved in cytotoxic drug metabolism are associated with an increased risk of FN [98]. In breast cancer patients treated with the FEC (5-FU + epirubicin + cyclophosphamide) regimen, *MDM2* SNP309 and *TP53* R72P genotypes [124], as well as SNPs of the *ABCC1/MRP1*, *UGT2B7*, and *FGFR4* genes [125], were significantly associated with an increased risk of FN. *UGT1A1* gene polymorphism is associated with increased risk of FN in patients treated with irinotecan [126,127,128]. Similarly, *DPYD* gene polymorphism resulting in dihydropyrimidine dehydrogenase (DPD) deficiency is associated with significant toxicity, including FN, in patients treated with fluoropyrimidines [127]. Prospective validation of these polymorphisms as predictive factors for FN could identify patients at high risk of infectious complications and suggest treatment deescalation or application of prophylactic measures in affected individuals [127].

## 7. Antibiotic Therapy in Patients with Solid Tumors

Early detection of infections is essential in cancer patients. Clinical signs of infection might be vague, especially in neutropenic patients, but fever remains an early, although non-specific, sign of infection [129]. Approximately 50–60% of patients who became febrile have an underlying infection [130]. However, non-infectious causes of fever, including paraneoplastic etiology (neoplastic fever) and drug reactions, are not rare in cancer patients [131] Moreover, laboratory markers of infection, including elevated C-reactive protein and leukocytosis, are frequently present in cancer patients [132,133], making the diagnosis of infection challenging. In such circumstances, additional markers such as procalcitonin can be used [134].

The symptoms-oriented diagnostic process should be initiated as soon as possible with identification of the most likely infection source. The empiric antibiotic should be initiated immediately after obtaining cultivation samples. The antibiotic choice should be tailored according to the suspected infection site, microbial colonization of the patient (i.e., known presence of multiresistant strains in previous cultivation samples), and the local epidemiological situation [10]. The most likely tumor-specific infections should be ruled out first, including cholangitis in pancreatic cancer, obstructive pneumonia in lung tumors, urinary infection in prostate cancer patients, etc. [135].

The use of antibiotics with broad-spectrum coverage, including anti-pseudomonal activity, is recommended in patients presenting with severe neutropenia [7]. Deescalation of the antibiotic therapy should be performed as soon as the results of cultivation samples are available, and the duration of antibiotic therapy should be tailored to the type of infection, level of immunosuppression, and other risk factors (type of malignancy, anticancer therapy, patient’s comorbidities, etc.). It should be kept in mind that prolonged antibiotic therapy can lead to severe complications, including vulvovaginal candidosis in women, *Clostridioides difficile* infection (CDI), and possible detrimental effects on anticancer therapy effectiveness due to dysmicrobia [136].

The rise in multiresistant strains of bacteria (including extended-spectrum β-lactamase [ESBL]-producing *Enterobacteriaceae*, methicillin-resistant *St. aureus* [MRSA], and vancomycin-resistant *Enterococcus faecium*) has become a significant problem, especially in oncologic patients [1]. Antimicrobial stewardship and strict adherence to infection control recommendations are essential to reduce the risk of emergence and spread of multiresistant bacteria strains [3]. However, approximately one third of antibiotics prescribed in the United States acute care hospitals are either unnecessary or suboptimal [137,138]. In up to 50% of cases, the treatment indication, choice of agent, or duration of antibiotic therapy may even be incorrect [139]. This is of upmost importance considering that 30-day mortality can reach 70% in patients with bloodstream infections not receiving appropriate antimicrobial treatment [140]. It is, therefore, the policy of many hospitals to restrict the use of broad-spectrum antibiotics unless approved by the hospital’s antibiotic center. This approach can reduce treatment expanses and limit the emergence of multiresistant strains and CDI [141,142].

The advent of modern treatment modalities, including targeted therapy and immunotherapy, has brought new challenges for the management of infections in cancer patients. Targeting checkpoints of immune response with ICIs has become a new treatment strategy in many solid tumors, including melanoma, renal cell carcinoma, lung cancer, and others. Despite great improvements in patients’ survival, a new class of adverse events, known as immune-related adverse events (irAE), has emerged [143]. This form of autoimmune reaction can affect various organs and be potentially life-threatening. Importantly, the discrimination between irAE and infectious complications may be challenging. This is of high clinical significance, considering that corticosteroids and other immunosuppressants used in irAE management may aggravate the course of infectious diseases and increase the risk of opportunistic infections [144]. Improper use of antibiotics in patients treated with ICIs may weaken the treatment outcome via antibiotic-induced dysbiosis [145]. Therefore, antibiotic therapy should be prescribed cautiously in patients treated with ICIs, and the diagnosis of infection should be confirmed with appropriate tests. Narrow-spectrum antibiotics are the preferred option in this setting, and consultation with the hospital’s antibiotic center before treatment initiation is highly encouraged [145].

## 8. Antibiotic Therapy of Specific Infections

### 8.1. Febrile Neutropenia

FN represents a severe and potentially life-threatening complication of cancer therapy, with an overall in-hospital mortality of ~10% [10]. The definition and risk factors for FN in patients with solid tumors have already been addressed. The management of FN follows international guidelines with slight variations reflecting national differences (i.e., local epidemiological situation and prevalence of multiresistant strains) [10,146].

The first step is to stratify patients into risk categories based on clinical characteristics (nature of the underlying malignancy, comorbidities, performance status, presence of hypotension, dehydration, and stress-induced hyperglycemia at presentation, outpatient status, and age). Evaluation of these characteristics allows us to stratify patients into low- and high-risk groups using the Multinational Association for Supportive Care in Cancer (MASCC) Risk Index Score [10] or Clinical Index of Stable Febrile Neutropenia (CISNE) score [147], the latter having been specifically validated for patients with solid tumors. FN patients within the high-risk group or having high-risk features as assessed by the admitting physician should be admitted to hospital and administered broad-spectrum antibiotics intravenously within one hour of admission (Table 3) [7,10]. Local epidemiological bacterial isolate and resistance patterns are crucial to determining the optimal empirical antibiotic therapy [10]. Inadequate antibiotic regimen use is associated with a significantly higher ICU admission and death rate during hospital stay [148]. Culture specimens, including two sets of blood cultures (from a peripheral vein and any indwelling venous catheter), urine specimens, and specimens from any suspected site of infection, should be obtained before antibiotic therapy initiation [7,10].

Patients at low risk of developing serious complications (<10%) may receive oral antibiotics in an outpatient setting after being provided careful patient education [146]. Outpatients should be instructed on how to monitor their symptoms and when and how to contact appropriate medical services. Compliance of the patient and ability to reach the healthcare facility are prerequisites for this approach [149].

**Table 3:** Appropriate initial antibiotics in febrile neutropenia groups with different risk of serious infection development.

Risk of Serious Complications	Low	High
Initial antibiotic	Oral or parenteral	Parenteral
	Inpatient or outpatient	Inpatient
	Amoxicillin-clavulanate + fluoroquinolone (ciprofloxacin or levofloxacin)	Antipseudomonal beta-lactam * (cefepime or meropenem or imipenem or piperacillin-tazobactam)
Suspicion of catheter-related infection, severe skin and soft tissue infection, pneumonia, or risk of MRSA infection	Shift to high-risk group	Add gram-positive bacteria targeted antibiotic (vancomycin or linezolid or daptomycin ^†^), in case of VRE add linezolid or daptomycin ^†^
Suspicion of abdominal infection	Shift to high-risk group	Add metronidazole
Risk of multiresistant strain infection	Shift to high-risk group	Choose carbapenem (in case of ESBL), add polymyxin-colistin or tigecycline (in cases of KPC)

ESBL–extended-spectrum beta-lactamase producing strains, KPC–carbapenemase producing strains, MRSA–methicillin-resistant *St. aureus,* VRE–vancomycin-resistant enterococci, * choice depends on the local epidemiological situation, ^†^ not in cases of pneumonia. References: [10,146,150,151].

### 8.2. Central Venous Catheter-Related Infections

CVC-related infections represent ~10% of bloodstream infections in cancer patients [152,153]. In case of suspected CVC-related infection, blood culture from the CVC and peripheral vein must be performed in order to determine differential time to positivity. A difference in time to positivity of >2 h (blood culture from CVC must be the first positive) is a highly sensitive and specific CVC-related infection indicator [154].

In particular situations, it might not be necessary to remove the infected CVC, and antimicrobial therapy alone is sufficient. The main premise for such an approach is a stable patient and the assumption of successful antimicrobial therapy without developing complications (Table 4) [155,156]. There is no consensus regarding the length of antimicrobial therapy. However, a recent meta-analysis suggests that a short-course therapy (seven days) for gram-negative bacteria, seven days for enterococci, and three days for coagulase-negative staphylococci could be sufficient in uncomplicated CVC-related infection [157]. However, the authors conclude that shorter courses may not be appropriate for immunocompromised patients, and prospective studies are warranted [157]. In case of complications, including tunnel infection, port abscess, septic thrombosis, endocarditis, and osteomyelitis, the catheter must always be removed, and appropriate pathogen-directed antimicrobial therapy should be used [155].

Another therapeutic option for stable patients with catheter-related infections caused by low-virulence pathogens—coagulase-negative staphylococci (except from *Staphylococcus lugdunensis*), *Corynebacterium* spp., and some gram-negative rods—is the use of antimicrobial lock therapy (ALT) either with or without delayed CVC removal [158]. This approach should only be used in combination with systemic administration of antibiotics [155,158]. The choice of antimicrobial agent depends on the type of isolated pathogen (Table 5). The dwell time of the lock solution differs according to the stability of the substance solution at the body temperature, but it should not exceed 48–72 h [158]. The type of CVC or port determines the volume of instilled antibiotic lock solution, which is ~2–5 mL. The most frequent antibiotics used for ALT are listed in Table 5. Appropriate duration of ALT is unknown, but generally 10–14 day therapy is recommended [155]. Importantly, the lock solution must be removed before catheter reuse.

Besides therapy for CVC-related infections, prophylactic ALT might decrease the incidence of central-line associated infections in cancer patients [159]. This approach seems promising and has demonstrated its cost-effectiveness specifically in cancer therapy [160], although further studies are warranted to optimize prophylactic ALT use.

**Table 5:** List of the most frequently used antibiotic catheter lock solutions.

Antibiotic	Spectrum of Bacteria	Concentration * (mg/mL)	Heparin Content (IU/mL)	Stability (Hours)	References
Vancomycin	gram-positive	2.0–5.0	2500 or 5000	72	[161,162]
Teicoplanin	gram-positive	5.0–10.0	0 or 100	96	[163,164]
Daptomycin	gram-positive	5.0	0 or 5000	72	[165]
Gentamicin	gram-positive, gram-negative	1.0–5.0	0, 2500 or 5000	72	[166,167]
Amikacin	gram-positive, gram-negative	1.0–40.0 ^†^	0 or 5000	72	[168]
Ceftazidime	gram-negative	0.5–10.0	0 or 5000	48	[167,169,170]
Cefazolin	Methicillin-sensitive staphylococci	5.0–10.0	2500 or 5000	72	[168]
Ciprofloxacin	gram-negative	0.2–5.0	0 or 5000	48	[171,172]
Ampicillin	Ampicillin-sensitive enterococci	10.0	10 or 5000	8 ^‡^	[161]
Ethanol	gram-positive, gram-negative	70%	0	24	[173]

* Concentration should exceed 100–1000× minimal inhibitory concentration (MIC). † Most commonly used 2.0 mg/mL, ‡ according to SPC [174]. For more detailed information on ACL see Justo et al. [168].

### 8.3. Pneumonia

Cancer patients are at increased risk of pneumonia due to impaired immune function caused by the tumor itself and cancer therapy [175], together with frequent tumor obstruction causing post-obstructive pneumonia [79]. The therapy of community-acquired pneumonia in cancer patients who are not neutropenic does not differ from that of the general population and should follow respective guidelines [7]. Beta-lactam antibiotics are the mainstay of therapy, but the choice depends on the local epidemiological situation (i.e., the local level of pneumococcal resistance). Antibiotics covering atypical pathogens (*Mycoplasma pneumoniae, Chlamydophila pneumoniae,* and *Legionella pneumophila*) are necessary for patients with community-acquired pneumonia [10]. In patients requiring hospital admission, respiratory fluoroquinolone or a combination of macrolide with a third generation cephalosporin (ceftriaxone or cefotaxime) or ertapenem are the options [7]. Besides gram-positive and gram-negative activity (excluding *Ps. aeruginosa* and *Acinetobacter* spp.), ertapenem has anaerobic activity useful for suspected aspiration and post-obstructive pneumonia [7,176]. Severe community-acquired pneumonia and pneumonia in neutropenic patients should be treated with a combination of an antipseudomonal beta-lactam (piperacillin/tazobactam) and a respiratory fluoroquinolone or azithromycin [7,10].

In patients who come from a medical facility or are long-term oncologically treated and repeatedly hospitalized, etiological microorganisms of pneumonia have changed, and therefore the empirical choice of antibiotic should be different, covering *St. aureus, Ps. aeruginosa,* or other gram-negative rods: piperacillin/tazobactam, cefepime, or carbapenems in combination with antibiotics covering gram-positive cocci (linezolid, vancomycin, or teicoplanin) [177]. In this regard, the choice of initial antibiotic regimen should be based on knowledge of the local patterns of antibiotic susceptibility [7,177]. Clinical samples, including good-quality sputum, lower respiratory tract samples for culture, viral (influenza, COVID-19), mycoplasma, chlamydial PCR detection, and urine samples for detection of pneumococcal and legionella antigens, should be obtained before antibiotic therapy initiation in case of severe pneumonia in immunocompromised patients [178,179]. Microbiological results can help to deescalate and target antibiotic treatment [177]. In patients receiving a prednisone equivalent of ≥20 mg for ≥4 months, or treated with RT and concomitant temozolomide without reliable antipneumocystis prophylaxis, the addition of high-dose trimethoprim/sulfamethoxazole should be considered [7,10].

### 8.4. Intra-Abdominal Infections

In addition to common intra-abdominal infections, cancer patients are at risk for infections complicating the underlying disease (infiltration of intra-abdominal organs by tumor, compression of adjacent organs by the tumor and associated stagnation of secretions, tumor disintegration with subsequent rupture, or creation of intra-abdominal or pelvic abscess) [180]. Antibiotic choice depends on the patient’s condition, previous antibiotic treatment, and colonization with multidrug-resistant bacteria. A high probability of polymicrobial pathogens and the presence of endogenous anaerobic flora has to be kept in mind [7]. Initial empirical treatment is based on administration of beta-lactam antibiotics (ampicillin/sulbactam, piperacillin/tazobactam, cefotaxime, ceftriaxone, carbapenem). If second-, third-, or fourth-generation cephalosporins are used, it is necessary to add metronidazole to cover anaerobic bacteria [10,180]. Antibiotic therapy with antipseudomonal activity is required in neutropenic patients [7]. Repeated cultivation of suitable materials (i.e., secretion from the drain, deep wound sample, and puncture fluid) may be required to avoid long-term administration of broad-spectrum antibiotics [180,181].

Cancer patients are susceptible to developing CDI as a result of the malignancy itself, immunosuppression, chemotherapy administration, antibiotic exposure, and frequent hospital stays [182]. This translates into a six- to nine-fold higher risk of developing CDI compared to non-cancer patients [183]. Orally administered fidaxomicin or vancomycin are the treatment of choice in initial and subsequent CDI episodes [184]. Orally administered metronidazole should be restricted to non-severe CDI cases when the above-mentioned agents are not available [184]. Fecal microbiota transplantation seems promising in the management of recurrent CDI, albeit the data in cancer patients are limited [7]. 

Neutropenic enterocolitis (referred to as typhlitis when located in the coecum) is not common in patients with solid tumors, but its association with taxanes (docetaxel, paclitaxel) and vinorelbine therapy has been reported [3]. Computed tomography is the preferred diagnostic tool for revealing thickening of the bowel wall [185]. The therapy consists of general supportive measures (including bowel rest and parenteral nutrition) and broad-spectrum antibiotics with coverage for *C. difficile*, aerobic pathogens, and anaerobic pathogens [3,7]. Complications develop in ~5% of patients requiring surgical intervention [186].

### 8.5. Urinary Tract Infections

UTIs are common in cancer patients due to cancer therapy, immunosuppression, and indwelling urinary catheters [187,188,189]. Importantly, the urinary tract is the source of ~20% of bloodstream infections in cancer patients [152,153]. The most common pathogens include *E. coli* (40–58%), *Kl. pneumoniae* (10–25%), *Ps. aeruginosa* (4–11%), *Enterococcus* spp. (8–11%), *Staphylococcus* spp. (11%), and *P. mirabilis* (1–5%) [187,188]. Multidrug resistance is frequent in cancer patients with UTIs (96%) [188]. A high proportion of resistance to fluroquinolones (90–96%), cephalosporins (68–80%), and aminoglycosides (46–50%) has been observed [189,190].

Uncomplicated UTIs (women without risk factors) can be treated according to general guidelines with orally administered fosfomycin, pivmecillinam, or nitrofurantoin [191]. Treatment with antimicrobials penetrating the prostate tissue (trimethoprim/sulfamethoxazole or fluoroquinolone) is required in male patients, provided cystitis can be associated with prostatitis [191]. However, most UTIs in oncological patients are associated with other risk factors, including anatomical or functional abnormalities of the urinary tract, indwelling urinary catheters, stents, renal disease, cancer-related immunosuppression, oncologic therapy, and surgical intervention [3,80]. Antimicrobial therapy should be guided by cultivation results, as repeated antibiotic therapy in cancer patients may lead to the selection of non-predictable bacterial variants and multiresistant strains [192]. Antimicrobial agents with extended spectrum of activity (piperacillin/tazobactam, imipenem, meropenem, ceftazidime/avibactam, ceftolozane/tazobactam) should be used for empirical treatment of severe infections in accordance with previous individual culture or local resistance data [191]. A switch to narrow-spectrum antibiotics should be performed as soon as the results of antibiotic sensitivity tests are available to avoid unnecessary adverse effects and ecological consequences [191,192]. Treatment for 7–14 days is generally recommended, but the duration should be guided by the therapy of the underlying abnormality [191,192].

## 9. The Impact of Antibiotic Therapy on Cancer Therapy Outcomes

Despite the undeniable contribution of antibiotic therapy to the management of infections in cancer patients, a potential detrimental effect on treatment outcome and toxicity has to be considered [193,194]. Several contributing factors have been proposed to decrease the efficacy of cancer therapy, the most prominent being alteration of the microbiome and its interaction with the patient’s immune system, potentially resulting in reduced immune surveillance [194,195].

### 9.1. Impact on Cancer Therapy Efficacy

There is a growing body of evidence that antibiotic therapy can negatively affect anticancer treatment efficacy and cancer-specific survival, especially in patients treated with immunotherapy [196]. It is now understood that the microbiome alters antitumor immunity and influences the efficacy of cancer therapies mediated via systemic immune response [194]. The effect of gut microbiome composition on immunotherapy outcome is supported by the finding that fecal transplants of gut microbiota from patients responding to ICI therapy to germ-free mice results in an antitumor response [197,198,199]. The presence of several species of microbiota have been associated with ICI efficacy, including *Bacteroides* spp. [200], *Bifidobacterium* spp. [198,201], *Faecalibacterium* spp. [202], *Akkermansia muciniphila* [199], *Collinsella aerofaciens* [198], *E. faecium* [198], and bacteria from the Ruminococcaceae family [197]. Changing the balance and diversity of the patient’s microbiome by antibiotic therapy may alter the antitumor immune response induced by ICIs, resulting in poor therapeutic outcomes [145]. Multiple clinical studies have reported a negative association between antibiotic use and response to ICIs in different solid tumors, including melanoma [203,204], NSCLC [203,205,206,207,208], and RCC [203,205,209]. Additionally, several meta-analyses have confirmed these results [210,211,212]. Interestingly, the negative effect of antibiotics on survival was also observed in adjuvant therapy with ICIs [204]. The most detrimental effect on overall survival in the multivariate analysis was observed in patients treated with penicillins, cephalosporins, and fluoroquinolones [204]. In light of these findings, the use of broad-spectrum antibiotics should be avoided in patients treated with immunotherapy whenever possible [195]. In addition to the antibiotic class, the length of antibiotic therapy seems to play an important role, as the detrimental effect was most commonly observed in patients receiving multiple or prolonged cycles of antibiotic therapy [203,207].

Besides affecting the efficacy of immunotherapy, the use of antibiotics can potentiate the effect of radiotherapy. Vancomycin, a glycopeptide antibiotic active against gram-positive bacteria with minimal absorption from the gut when administered orally, showed a potentiating antitumor effect when combined with radiotherapy in a preclinical model [213]. The effect was mediated through changes in gut microbiota composition, which led to increased antigen presentation by CD11c^+^ dendritic cells in the tumor-draining lymph nodes of the radiotherapy-treated mice. Interestingly, the vancomycin effect was abrogated by butyrate, a metabolite produced by vancomycin-depleted gut bacteria [213]. Recently, these results were confirmed by another study, suggesting that butyrate-producing bacteria, such as Lachnospiraceae and Ruminococcaceae, could be novel therapeutic targets [214].

Conversely, antibiotic therapy during curative chemoradiotherapy in patients with locally advanced head and neck cancers was associated with a significant reduction of progression-free survival, overall survival, and disease-specific survival [215]. The potential harm of broad-spectrum and prophylactic antibiotic therapy in these patients should, therefore, be considered [215].

### 9.2. Impact on Cancer Therapy Toxicity

In addition to possible pharmacokinetic interactions, the use of antibiotics can lead to increased toxicity of anticancer drugs by modulation of microbiome [195]. In patients with metastatic pancreatic adenocarcinoma treated with gemcitabine, antibiotic therapy was associated with increased chemotherapy-related toxicity [216]. The authors suggest that intratumor bacteria may be responsible for a clinically meaningful portion of gemcitabine metabolism [216]. In melanoma patients treated with immunotherapy, the use of antibiotics was associated with moderate to severe immune-mediated colitis [204]. The underlying mechanism seems to involve changes in the gut microbiome, resulting in inhibition of regulatory T cells [217] together with regrowth of pro-inflammation bacterial species [204].

## 10. Conclusions

Infections are one of the most common causes of death in patients with solid tumors and can complicate cancer therapy. Thorough evaluation of possible risk factors could identify patients at high risk for infections and aid in the decision-making process regarding the use of G-CSF and antibiotics. In this regard, prognostic scores considering multiple risk factors together are being evaluated to calculate each individual patient’s risk. Early initiation of antibiotic therapy is essential, especially in immunocompromised patients. However, other pathologic conditions mimicking infection, including irAE in patients treated with ICIs, have to be excluded first. Consultation with the hospital’s antibiotic center before antibiotic therapy initiation and strict adherence to the antibiotic stewardship program is highly encouraged to reduce the occurrence of multidrug-resistant pathogens and decrease therapy costs. Potential risks of antibiotic therapy in cancer patients have to be considered, including potential detrimental effects on treatment efficacy and toxicity. This holds especially true for patients treated with ICIs, for whom antibiotic therapy should be restricted to unequivocally diagnosed infections, and broad-spectrum antibiotics should be avoided.

## Figures and Tables

**Table 1:** Selected chemotherapy regimens with a significant risk of febrile neutropenia.

Tumor Type	Chemotherapy Regimen	Risk of FN (%)	Reference
Breast cancer	AC (Doxorubicin/Cyclophosphamide)	7–13 *	Truong et al. [13]
	AC⟶D (Doxorubicin/Cyclophosphamide⟶Docetaxel)	25	Perez et al. [14]
	TAC (Docetaxel/Doxorubicin/Cyclophosphamide)	22	Von Minckwitz et al. [15]
	TC (Docetaxel/Cyclophosphamide)	70	Kosaka et al. [16]
	TCH (Docetaxel/Carboplatin/Trastuzumab)	41	Gilbar et al. [17]
	Docetaxel	17	Marty et al. [18]
Bladder cancer	MVAC (Methotrexate/Vinblastine/Doxorubicin/Cisplatin)	26	Sternberg et al. [19]
Cervical cancer	Cisplatin/Paclitaxel	28	Rose et al. [20]
	Cisplatin/Topotecan	18	Long et al. [21]
Gastric cancer	DCF (Docetaxel/Cisplatin/5-FU) ^†^	29	Van Cutsem et al. [22]
	TCF (Docetaxel/Cisplatin/5-FU) ^‡^	41	Roth et al. [23]
	ECF (Epirubicin/Cisplatin/5-FU)	13–18	Roth et al. [23], Cunningham et al. [24]
	ECX (Epirubicin/Cisplatin/Capecitabine)	11	Cunningham et al. [24]
Germ cell tumors	BEP (Bleomycin/Etoposide/Cisplatin)	13	Fossa et al. [25]
	EP (Etoposide/Cisplatin)	10	Motzer et al. [26]
	VIP (Etoposide/Ifosfamide/Cisplatin)	15	Fujiwara et al. [27]
	VeIP (Vinblastine/Etoposide/Cisplatin)	67	Miller et al. [28]
	TIP (Paclitaxel/Ifosfamide/Cisplatin)	48	Kondagunta et al. [29]
HNSCC	TPF (Docetaxel/Cisplatin/5-FU)	11	Pointreau et al. [30]
NSCLC	Cisplatin/Paclitaxel	16	Schiller et al. [31]
	Cisplatin/Vinorelbine	22	Pujol et al. [32]
	Cisplatin/Docetaxel	5–11	Fossella et al. [33], Schiller et al. [31]
	Cisplatin/Etoposide	54 ^§^ 12 ^¶^	Font et al. [34] Cardenal et al. [35]
	Docetaxel/Carboplatin	26	Millward et al. [36]
Ovarian cancer	Topotecan	42	Swisher et al. [37]
	Docetaxel	33	Verschraegen et al. [38]
	Paclitaxel	22	Omura et al. [39]
Pancreatic cancer	FOLFIRINOX (5-FU/Leucovorin/Oxaliplatin/Irinotecan)	17	Hosein et al. [40]
SCLC	Etoposide/Carboplatin	14	Yilmaz et al. [41]
	Topotecan	28	Von Pawel et al. [42]
	ICE (Ifosfamide/Carboplatin/Etoposide)	24	Lorigan et al. [43]
	CAV (Cyclophosphamide/Doxorubicin/Vincristine)	14	White et al. [44]
Soft tissue sarcoma	MAID (Mesna/Doxorubicin/Ifosfamide/Dacarbazin)	58	Binh Nguyen et al. [45]
	Ifosfamide	18 ^#^, 20 ^##^	Lorigan et al. [46]

5-FU–5-fluorouracil, HNSCC–Head and neck squamous cell carcinoma, NSCLC–Non-small cell lung cancer, SCLC–Small cell lung cancer, * Results from a systematic meta-analysis (randomized controlled trials and observational studies, respectively), ^†^ Docetaxel 75 mg/m^2^ D1 + Cisplatin 75 mg/m^2^ D1 + 5-FU 750 mg/m^2^/day D1–5 q3w, ^‡^ Docetaxel 85 mg/m^2^ D1 + Cisplatin 75 mg/m^2^ D1 + 5-FU 300 mg/m^2^/day D1–14, ^§^ Cisplatin 35 mg/m^2^ D1–3 + Etoposide 200 mg/m^2^ D1–3, ^¶^ Cisplatin 100 mg/m^2^ D1 + Etoposide 100 mg/m^2^ D1–3, ^#^ Ifosfamide 3 g/m^2^ 3-h infusion D1–3, ^##^ Ifosfamide 9 g/m^2^ continuous D1–3. For more information, see Aapro et al. (2011) [11].

**Table 2:** Independent risk factors for FN in solid tumors (risk score model studies).

Reference	Study Population	Risk Factors (Multivariate Analysis)
Aagaard et al. (2018) [57]	Patients with solid tumors and DLBCL treated with first-line chemotherapy (*n* = 9458)	Female sex, age > 65 years, cancer type, disease stage, low albumin, elevated bilirubin, low estimated glomerular filtration rate, infection before baseline, treatment with more than one chemotherapy drug (two to four), receiving taxane-based chemotherapy
Aagaard et al. (2020) [59]	Patients with solid tumors who initiated cycle 2 of standard first-line chemotherapy (*n* = 6885)	Higher predicted risk for FN in the first cycle, platinum- and taxane-containing regimens, concurrent radiotherapy, treatment in cycle 2 compared to later cycles, previous FN or neutropenia, not receiving G-CSF
Hosmer et al. (2011) [55]	Elderly patients with breast, lung, colorectal, and prostate cancer (*n* = 58,053)	Advanced age at diagnosis, number of associated comorbid conditions, receipt of immunosuppressive chemotherapy, receipt of chemotherapy within one month of diagnosis
Lyman et al. (2011) [56] *	Patients with breast, lung, colorectal, ovarian cancer, and lymphoma patients (*n* = 4458)	Prior chemotherapy, use of other immunosuppressive medications, abnormal hepatic and renal function, low white blood count, chemotherapy and planned delivery ≥ 85%, small cell lung cancer, specific classes of chemotherapy (anthracyclines, taxanes, certain alkylating agents [cyclophosphamide, ifosfamide], class I/II topoisomerase inhibitors, platinum derivates [cisplatin, carboplatin], gemcitabine, vinorelbine)
Razzaghdoust et al. (2018) [58] *	Patients with various solid tumors and lymphomas (*n* = 305)	High-risk chemotherapy regimen without G-CSF, intermediate-risk regimen without G-CSF, age > 65 years, elevated ferritin, BMI < 1.73 kg/m^2^, BSA < 2 m^2^, estimated glomerular filtration rate < 60 mL/min/1.73 m^2^, elevated C-reactive protein

* prospectively validated studies, DLBCL–Diffuse large B-cell lymphoma, FN–febrile neutropenia, G-CSF–Granulocyte colony-stimulating factors, BMI–body mass index, BSA–body surface area.

**Table 4:** Appropriate antibiotic treatment of bacterial CVC-related infection according to isolated pathogens.

Isolated Pathogen	Catheter Removal	Antibiotic Therapy	
		Choice	Duration
Coagulase-negative staphylococci	Not necessary Risk factor for recurrence	Vancomycin Oxacillin * Flucloxacillin * Cefazoline *	Catheter removed: 5–7 days Retained catheter: 10–14 days + ALT 10–14 days
*St. aureus, St. lugdunensis*	Yes	Vancomycin Oxacillin * Flucloxacillin * Cefazoline *	≥14 days. Necessary to rule out complications. Complications: 4–6 weeks
Enterococci	Yes Long-term CVC may retain	Vancomycin Ampicillin *	5–14 days. Retained long-term CVC: 7–14 days + ALT 7–14 days
gram-negative bacilli	Yes, especially in case of multiresistant bacteria CVC retaining unsuitable for immunosuppressed patients	Based on severity of disease: Piperacillin/tazobactam *, 4th gen. Cephalosporin *, Carbapenem +/- Aminoglycoside *	7–14 days

* According to sensitivity pattern and local susceptibility data. CVC–central venous catheter, ALT–antimicrobial lock therapy. References: [155,156].

## Data Availability

Not applicable.

## References

[1] Gudiol C., Aguado J.M., Carratalà J. (2016). Bloodstream infections in patients with solid tumors. Virulence.

[2] Safdar A., Armstrong D. (2001). Infectious morbidity in critically ill patients with cancer. Crit. Care Clin..

[3] Rolston K.V.I. (2017). Infections in Cancer Patients with Solid Tumors: A Review. Infect. Dis. Ther..

[4] Williams M.D., Braun L.A., Cooper L.M., Johnston J., Weiss R.V., Qualy R.L., Linde-Zwirble W. (2004). Hospitalized cancer patients with severe sepsis: Analysis of incidence, mortality, and associated costs of care. Crit. Care.

[5] Schelenz S., Nwaka D., Hunter P.R. (2013). Longitudinal surveillance of bacteraemia in haematology and oncology patients at a UK cancer centre and the impact of ciprofloxacin use on antimicrobial resistance. J. Antimicrob. Chemother..

[6] Zheng Y., Chen Y., Yu K., Yang Y., Wang X., Yang X., Qian J., Liu Z.-X., Wu B. (2021). Fatal Infections among Cancer Patients: A Population-Based Study in the United States. Infect. Dis. Ther..

[7] National Comprehensive Cancer Network (2021). NCCN Clinical Practice Guidelines in Oncology. Prevention and Treatment of Cancer-Related Infections 1. https://www.nccn.org/professionals/physician_gls/pdf/infections.pdf.

[8] Marin M., Gudiol C., Ardanuy C., Garcia-Vidal C., Calvo M., Arnan M., Carratalà J. (2014). Bloodstream infections in neutropenic patients with cancer: Differences between patients with haematological malignancies and solid tumours. J. Infect..

[9] Fillatre P., Decaux O., Jouneau S., Revest M., Gacouin A., Robert-Gangneux F., Fresnel A., Guiguen C., Le Tulzo Y., Jégo P. (2014). Incidence of Pneumocystis jiroveci Pneumonia among Groups at Risk in HIV-negative Patients. Am. J. Med..

[10] Klastersky J., de Naurois J., Rolston K., Rapoport B., Maschmeyer G., Aapro M., Herrstedt J. (2016). Management of febrile neutropaenia: ESMO Clinical Practice Guidelines. Ann. Oncol..

[11] Aapro M., Bohlius J., Cameron D., Lago L.D., Donnelly J.P., Kearney N., Lyman G., Pettengell R., Tjan-Heijnen V., Walewski J. (2011). 2010 update of EORTC guidelines for the use of granulocyte-colony stimulating factor to reduce the incidence of chemotherapy-induced febrile neutropenia in adult patients with lymphoproliferative disorders and solid tumours. Eur. J. Cancer.

[12] De Miguel S.C., Calleja-Hernández M., Menjón-Beltrán S., Vallejo-Rodríguez I. (2015). Granulocyte colony-stimulating factors as prophylaxis against febrile neutropenia. Support. Care Cancer.

[13] Truong L.D., Shen S.S. (2011). Immunohistochemical diagnosis of renal neoplasms. Arch. Pathol. Lab. Med..

[14] Perez E.A., Geeraerts L., Suman V.J., Adjei A.A., Baron A.T., Hatfield A.K., Maihle N., Michalak J.C., Kuross S.A., Kugler J.W. (2002). A randomized phase II study of sequential docetaxel and doxorubicin/cyclophosphamide in patients with metastatic breast cancer. Ann. Oncol..

[15] von Minckwitz G., Schneeweiss A., Loibl S., Salat C., Denkert C., Rezai M., Blohmer J.U., Jackisch C., Paepke S., Gerber B. (2014). Neoadjuvant carboplatin in patients with triple-negative and HER2-positive early breast cancer (GeparSixto; GBG 66): A randomised phase 2 trial. Lancet Oncol..

[16] Kosaka Y., Rai Y., Masuda N., Takano T., Saeki T., Nakamura S., Shimazaki R., Ito Y., Tokuda Y., Tamura K. (2015). Phase III placebo-controlled, double-blind, randomized trial of pegfilgrastim to reduce the risk of febrile neutropenia in breast cancer patients receiving docetaxel/cyclophosphamide chemotherapy. Support. Care Cancer.

[17] Gilbar P., McPherson I., Sorour N., Sanmugarajah J. (2014). High incidence of febrile neutropenia following adjuvant breast chemotherapy with docetaxel, carboplatin and trastuzumab. Breast Cancer Manag..

[18] Marty M., Cognetti F., Maraninchi D., Snyder R., Mauriac L., Tubiana-Hulin M., Chan S., Grimes D., Antón A., Lluch A. (2005). Randomized phase II trial of the efficacy and safety of trastuzumab combined with docetaxel in patients with human epidermal growth factor receptor 2–Positive metastatic breast cancer administered as first-line treatment: The M77001 study group. J. Clin. Oncol..

[19] Sternberg C.N., De Mulder P.H., Schornagel J.H., Théodore C., Fossa S.D., Van Oosterom A.T., Witjes F., Spina M., Van Groeningen C.J., De Balincourt C. (2001). Randomized phase III trial of high-dose-intensity methotrexate, vinblastine, doxorubicin, and cisplatin (MVAC) chemo-therapy and recombinant human granulocyte colony-stimulating factor versus classic MVAC in advanced urothelial tract tumors: European Organization for Research and Treatment of Cancer Protocol no. 30924. J. Clin. Oncol..

[20] Rose P.G., Blessing J.A., Gershenson D.M., McGehee R. (1999). Paclitaxel and cisplatin as first-line therapy in recurrent or advanced squamous cell carcinoma of the cervix: A gynecologic oncology group study. J. Clin. Oncol..

[21] Long H.J., Bundy B.N., Grendys E.C., Benda J.A., McMeekin D.S., Sorosky J., Miller D., Eaton L.A., Fiorica J.V. (2005). Randomized Phase III Trial of Cisplatin with or without Topotecan in Carcinoma of the Uterine Cervix: A Gynecologic Oncology Group Study. J. Clin. Oncol..

[22] Van Cutsem E., Moiseyenko V., Tjulandin S., Majlis A., Constenla M., Boni C., Rodrigues A., Fodor M., Chao Y., Voznyi E. (2006). Phase III study of docetaxel and cisplatin plus fluorouracil compared with cisplatin and fluorouracil as first-line therapy for advanced gastric cancer: A report of the v325 study group. J. Clin. Oncol..

[23] Roth A.D., Fazio N., Stupp R., Falk S., Bernhard J., Saletti P., Köberle D., Borner M.M., Rufibach K., Maibach R. (2007). Docetaxel, cisplatin, and fluorouracil; Docetaxel and cisplatin; and epirubicin, cisplatin, and fluorouracil as systemic treatment for advanced gastric carcinoma: A randomized phase II trial of the swiss group for clinical cancer research. J. Clin. Oncol..

[24] Cunningham D., Starling N., Rao S., Iveson T., Nicolson M., Coxon F., Middleton G., Daniel F., Oates J., Norman A.R. (2008). Capecitabine and oxaliplatin for advanced esophagogastric cancer. N. Engl. J. Med..

[25] Fossa S.D., Kaye S.B., Mead G.M., Cullen M., De Wit R., Bodrogi I., Van Groeningen C.J., De Mulder P.H., Stenning S., Lallemand E. (1998). Filgrastim during combination chemotherapy of patients with poor-prognosis metastatic germ cell malignancy. J. Clin. Oncol..

[26] Motzer R.J., Sheinfeld J., Mazumdar M., Bajorin D.F., Bosl G.J., Herr H., Lyn P., Vlamis V. (1995). Etoposide and cisplatin adjuvant therapy for patients with pathologic stage II germ cell tumors. J. Clin. Oncol..

[27] Fujiwara M., Tanaka H., Yuasa T., Komai Y., Oguchi T., Fujiwara R., Numao N., Yamamoto S., Fujii Y., Fukui I. (2021). First-Line combination chemotherapy with etoposide, ifosfamide and cisplatin for the treatment of disseminated germ cell cancer: Efficacy and feasibility in current clinical practice. Int. J. Urol..

[28] Miller K.D., Loehrer P.J., Gonin R., Einhorn L.H. (1997). Salvage chemotherapy with vinblastine, ifosfamide, and cisplatin in recurrent seminoma. J. Clin. Oncol..

[29] Kondagunta G.V., Bacik J., Donadio A., Bajorin D., Marion S., Sheinfeld J., Bosl G.J., Motzer R.J. (2005). Combination of paclitaxel, ifosfamide, and cisplatin is an effective second-line therapy for patients with relapsed testicular germ cell tumors. J. Clin. Oncol..

[30] Pointreau Y., Garaud P., Chapet S., Sire C., Tuchais C., Tortochaux J., Faivre S., Guerrif S., Alfonsi M., Calais G. (2009). Randomized trial of induction chemotherapy with cisplatin and 5-fluorouracil with or without docetaxel for larynx preservation. J. Natl. Cancer Inst..

[31] Schiller J.H., Harrington D., Belani C., Langer C., Sandler A., Krook J., Zhu J., Johnson D.H. (2002). Comparison of four chemotherapy regimens for advanced non–small-cell lung cancer. N. Engl. J. Med..

[32] Pujol J.-L., Breton J.-L., Gervais R., Rebattu P., Depierre A., Morère J.-F., Milleron B., Debieuvre D., Castéra D., Souquet P.-J. (2005). Gemcitabine–Docetaxel versus cisplatin–vinorelbine in advanced or metastatic non-small-cell lung cancer: A phase III study addressing the case for cisplatin. Ann. Oncol..

[33] Fossella F., Pereira J.R., Von Pawel J., Pluzanska A., Gorbounova V., Kaukel E., Mattson K.V., Ramlau R., Szczęsna A., Fidias P. (2003). Randomized, Multinational, Phase III Study of Docetaxel Plus Platinum Combinations Versus Vinorelbine Plus Cisplatin for Advanced Non–Small-Cell Lung Cancer: The TAX 326 Study Group. J. Clin. Oncol..

[34] Font A., Moyano A.J., Puerto J.M., Tres A., Garcia-Giron C., Barneto I., Anton A., Sanchez J.J., Salvador A., Rosell R. (1999). Increasing dose intensity of cisplatin-etoposide in advanced nonsmall cell lung carcinoma. A phase III randomized trial of the spanish lung cancer group. Cancer.

[35] Cardenal F., López-Cabrerizo M.P., Antón A., Alberola V., Massuti B., Carrato A., Barneto I., Lomas M., García M., Lianes P. (1999). Randomized phase III study of gemcitabine-cisplatin versus etoposide-cisplatin in the treatment of locally advanced or metastatic non-small-cell lung cancer. J. Clin. Oncol..

[36] Millward M.J., Boyer M.J., Lehnert M., Clarke S., Rischin D., Goh B.-C., Wong J., McNeil E., Bishop J.F. (2003). Docetaxel and carboplatin is an active regimen in advancednon-small-cell lung cancer: A phase II study in Caucasian and Asian patients. Ann. Oncol..

[37] Swisher E.M., Mutch D.G., Rader J.S., Elbendary A., Herzog T.J. (1997). Topotecan in platinum- and paclitaxel-resistant ovarian cancer. Gynecol. Oncol..

[38] Verschraegen C.F., Sittisomwong T., Kudelka A.P., Guedes E.D.P., Steger M., Nelson-Taylor T., Vincent M., Rogers R., Atkinson E.N., Kavanagh J.J. (2000). Docetaxel for Patients With Paclitaxel-Resistant Müllerian Carcinoma. J. Clin. Oncol..

[39] Omura G.A., Brady M.F., Look K.Y., Averette H.E., Delmore J.E., Long H.J., Wadler S., Spiegel G., Arbuck S.G. (2003). Phase III trial of paclitaxel at two dose levels, the higher dose accompanied by filgrastim at two dose levels in platinum-pretreated epithelial ovarian cancer: An intergroup study. J. Clin. Oncol..

[40] Hosein P.J., MacIntyre J., Kawamura C., Maldonado J.C., Ernani V., Loaiza-Bonilla A., Narayanan G., Ribeiro A., Portelance L., Merchan J.R. (2012). A retrospective study of neoadjuvant FOLFIRINOX in unresectable or borderline-resectable locally advanced pancreatic adenocarcinoma. BMC Cancer.

[41] Yilmaz U., Anar C., Polat G., Halilcolar H. (2011). Carboplatin plus etoposide for extensive stage small-cell lung cancer: An experience with AUC 6 doses of carboplatin. Indian J. Cancer.

[42] Von Pawel J., Schiller J.H., Shepherd F.A., Fields S.Z., Kleisbauer J., Chrysson N.G., Stewart D.J., Clark P.I., Palmer M.C., De Pierre A. (1999). Topotecan versus cyclophosphamide, doxorubicin, and vincristine for the treatment of recurrent small-cell lung cancer. J. Clin. Oncol..

[43] Lorigan P., Woll P., O’Brien M.E.R., Ashcroft L.F., Sampson M.R., Thatcher N. (2005). Randomized phase III trial of dose-dense chemotherapy supported by whole-blood hematopoietic progenitors in better-prognosis small-cell lung cancer. J. Natl. Cancer Inst..

[44] White S.C., Lorigan P., Middleton M.R., Anderson H., Valle J., Summers Y., Burt P.A., Arance A., Stout R., Thatcher N. (2001). Randomized phase II study of cyclophosphamide, doxorubicin, and vincristine compared with single-agent carboplatin in patients with poor prognosis small cell lung carcinoma. Cancer.

[45] Bui B.N., Chevallier B., Chevreau C., Krakowski I., Peny A.M., Thyss A., Maugard-Louboutin C., Cupissol D., Fargeot P., Bonichon F. (1995). Efficacy of lenograstim on hematologic tolerance to MAID chemotherapy in patients with advanced soft tissue sarcoma and consequences on treatment dose-intensity. J. Clin. Oncol..

[46] Lorigan P., Verweij J., Papai Z., Rodenhuis S., Le Cesne A., Leahy M., Radford J., Van Glabbeke M.M., Kirkpatrick A., Hogendoorn P. (2007). Phase III trial of two investigational schedules of ifosfamide compared with standard-dose doxorubicin in advanced or metastatic soft tissue sarcoma: A european organisation for research and treatment of cancer soft tissue and bone sarcoma group study. J. Clin. Oncol..

[47] Thomson A.W., Turnquist H.R., Raimondi G. (2009). Immunoregulatory functions of mTOR inhibition. Nat. Rev. Immunol..

[48] Kaymakcalan M., Je Y., Sonpavde G., Galsky M., Nguyen P.L., Heng D.Y.C., Richards C.J., Choueiri T.K. (2013). Risk of infections in renal cell carcinoma (RCC) and non-RCC patients treated with mammalian target of rapamycin inhibitors. Br. J. Cancer.

[49] Alvarez R.H., Bechara R.I., Naughton M.J., Adachi J.A., Reuben J.M. (2018). Emerging perspectives on mtor inhibitor-associated pneumonitis in breast cancer. Oncologist.

[50] Maschmeyer G., De Greef J., Mellinghoff S.C., Nosari A., Thiebaut-Bertrand A., Bergeron A., Franquet T., Blijlevens N.M.A., Maertens J.A., on behalf of the European Conference on Infections in Leukemia (2019). Infections associated with immunotherapeutic and molecular targeted agents in hematology and oncology. A position paper by the European Conference on Infections in Leukemia (ECIL). Leukemia.

[51] Finn R.S., Aleshin A., Slamon D.J. (2016). Targeting the cyclin-dependent kinases (CDK) 4/6 in estrogen receptor-positive breast cancers. Breast Cancer Res..

[52] Hu W., Sung T., Jessen B.A., Thibault S., Finkelstein M.B., Khan N.K., Sacaan A.I. (2016). Mechanistic Investigation of Bone Marrow Suppression Associated with Palbociclib and its Differentiation from Cytotoxic Chemotherapies. Clin. Cancer Res..

[53] Gelbert L.M., Cai S., Lin X., Sanchez-Martinez C., Del Prado M., Lallena M.J., Torres R., Ajamie R.T., Wishart G.N., Flack R.S. (2014). Preclinical characterization of the CDK4/6 inhibitor LY2835219: In-Vivo cell cycle-dependent/independent anti-tumor activities alone/in combination with gemcitabine. Investig. N. Drugs.

[54] Som A., Mandaliya R., Alsaadi D., Farshidpour M., Charabaty A., Malhotra N., Mattar M.C. (2019). Immune checkpoint inhibitor-induced colitis: A comprehensive review. World J. Clin. Cases.

[55] Hosmer W., Malin J., Wong M. (2011). Development and validation of a prediction model for the risk of developing febrile neutropenia in the first cycle of chemotherapy among elderly patients with breast, lung, colorectal, and prostate cancer. Support. Care Cancer.

[56] Lyman G.H., Kuderer N.M., Crawford J., Wolff D.A., Culakova E., Poniewierski M.S., Dale D.C. (2011). Predicting individual risk of neutropenic complications in patients receiving cancer chemotherapy. Cancer.

[57] Aagaard T., Roen A., Reekie J., Daugaard G., Brown P.D.N., Specht L., Sengeløv H., Mocroft A., Lundgren J., Helleberg M. (2018). Development and validation of a risk score for febrile neutropenia after chemotherapy in patients with cancer: The FENCE score. JNCI Cancer Spectr..

[58] Razzaghdoust A., Mofid B., Moghadam M. (2018). Development of a simplified multivariable model to predict neutropenic complications in cancer patients undergoing chemotherapy. Support. Care Cancer.

[59] Aagaard T., Reekie J., Roen A., Daugaard G., Specht L., Sengeløv H., Mocroft A., Lundgren J., Helleberg M. (2020). Development and validation of a cycle-specific risk score for febrile neutropenia during chemotherapy cycles 2–6 in patients with solid cancers: The CSR FENCE score. Int. J. Cancer.

[60] NCI Common Terminology Criteria for Adverse Events (CTCAE) Version 4.0. https://evs.nci.nih.gov/ftp1/CTCAE/CTCAE_4.03/CTCAE_4.03_2010-06-14_QuickReference_8.5x11.pdf.

[61] Bodey G.P., Buckley M., Sathe Y.S., Freireich E.J. (1966). Quantitative relationships between circulating leukocytes and infection in patients with acute leukemia. Ann. Intern. Med..

[62] Castagnola E., Mikulska M., Viscoli C., Bennett J.E., Dolin R., Blaser M.J. (2015). Prophylaxis and Empirical Therapy of Infection in Cancer Patients. Mandell, Douglas, and Bennett’s Principles and Practice of Infectious Diseases.

[63] Nishimura N., Yamada S., Ueda K., Mishima Y., Yokoyama M., Saotome T., Terui Y., Takahashi S., Hatake K., Nishimura M. (2010). Incidence and severity of oral mucositis induced by conventional chemotherapy: A comprehensive prospective analysis of 227 cancer patients. J. Clin. Oncol..

[64] Elting L.S., Chang Y.-C., Parelkar P., Boers-Doets C.B., Michelet M., Hita G., Rouleau T., Cooksley C., Halm J., Vithala M. (2013). Risk of oral and gastrointestinal mucosal injury among patients receiving selected targeted agents: A meta-analysis. Support. Care Cancer.

[65] Kwitkowski V.E., Prowell T.M., Ibrahim A., Farrell A.T., Justice R., Mitchell S.S., Sridhara R., Pazdur R. (2010). FDA Approval Summary: Temsirolimus as Treatment for Advanced Renal Cell Carcinoma. Oncologist.

[66] Peterson D.E., Boers-Doets C., Bensadoun R.J., Herrstedt J. (2015). Management of oral and gastrointestinal mucosal injury: ESMO Clinical Practice Guidelines for diagnosis, treatment, and follow-up. Ann. Oncol..

[67] Böll B., Schalk E., Buchheidt D., Hasenkamp J., Kiehl M., Kiderlen T.R., Kochanek M., Koldehoff M., Kostrewa P., Claßen A.Y. (2021). Central venous catheter–related infections in hematology and oncology: 2020 updated guidelines on diagnosis, management, and prevention by the Infectious Diseases Working Party (AGIHO) of the German Society of Hematology and Medical Oncology (DGHO). Ann. Hematol..

[68] Taxbro K., Hammarskjöld F., Thelin B., Lewin F., Hagman H., Hanberger H., Berg S. (2019). Clinical impact of peripherally inserted central catheters vs implanted port catheters in patients with cancer: An open-label, randomised, two-centre trial. Br. J. Anaesth..

[69] Pu Y.-L., Li Z.-S., Zhi X.-X., Shi Y.-A., Meng A.-F., Cheng F., Ali A., Li C., Fang H., Wang C. (2020). Complications and costs of peripherally inserted central venous catheters compared with implantable port catheters for cancer patients. Cancer Nurs..

[70] Corti F., Brambilla M., Manglaviti S., Di Vico L., Pisanu M.N., Facchinetti C., Dotti K.F., Lanocita R., Marchianò A., De Braud F. (2021). Comparison of outcomes of central venous catheters in patients with solid and hematologic neoplasms: An Italian real-world analysis. Tumori J..

[71] Dezfulian C., Lavelle J., Nallamothu B.K., Kaufman S.R., Saint S. (2003). Rates of infection for single-lumen versus multilumen central venous catheters: A meta-analysis. Crit. Care Med..

[72] Bouza E., Burillo A., Muñoz P. (2002). Catheter-related infections: Diagnosis and intravascular treatment. Clin. Microbiol. Infect..

[73] Wisplinghoff H., Seifert H., Wenzel R.P., Edmond M. (2003). Current trends in the epidemiology of nosocomial bloodstream infections in patients with hematological malignancies and solid neoplasms in hospitals in the united states. Clin. Infect. Dis..

[74] Marcos M., Soriano A., Iñurrieta A., Martínez J.A., Romero A., Cobos N., Hernández C., Almela M., Marco F., Mensa J. (2011). Changing epidemiology of central venous catheter-related bloodstream infections: Increasing prevalence of Gram-negative pathogens. J. Antimicrob. Chemother..

[75] Chaftari A.M., Hachem R., Jiang Y., Shah P., Hussain A., Al Hamal Z., Yousif A., Jordan M., Michael M., Raad I. (2018). Changing Epidemiology of Catheter-Related Bloodstream Infections in Cancer Patients. Infect. Control. Hosp. Epidemiol..

[76] Abers M.S., Sandvall B.P., Sampath R., Zuno C., Uy N., Yu V.L., Stager C.E., Musher D.M. (2016). Postobstructive pneumonia: An underdescribed syndrome. Clin. Infect. Dis..

[77] Rolston K.V. (2016). Postobstructive pneumonia in cancer patients. Clin. Infect. Dis..

[78] Kalkat M.S., Bonser R.S. (2003). Obstructive pneumonia: An indication for surgery in mega aorta syndrome. Ann. Thorac. Surg..

[79] Rolston K.V.I., Nesher L. (2018). Post-Obstructive pneumonia in patients with cancer: A review. Infect. Dis. Ther..

[80] Seo S.K., Liu C., Dadwal S.S. (2021). Infectious disease complications in patients with cancer. Crit. Care Clin..

[81] Battaglia C.C., Hale K. (2018). Hospital-Acquired infections in critically III patients with cancer. J. Intensive Care Med..

[82] Bahu R., Chaftari A.-M., Hachem R.Y., Ahrar K., Shomali W., El Zakhem A., Jiang Y., AlShuaibi M., Raad I.I. (2013). Nephrostomy tube related pyelonephritis in patients with cancer: Epidemiology, infection rate and risk factors. J. Urol..

[83] Pu L.Z.C.T., Singh R., Loong C.K., de Moura E.G.H. (2016). Malignant Biliary Obstruction: Evidence for Best Practice. Gastroenterol. Res. Pract..

[84] Cassani L., Lee J.H. (2015). Management of malignant distal biliary obstruction. Gastrointest. Interv..

[85] Shi S., Xia W., Guo H., Kong H., Zheng S. (2016). Unique characteristics of pyogenic liver abscesses of biliary origin. Surgery.

[86] Rolston K.V.I., Dholakia N., Rodriguez S., Rubenstein E.B. (1995). Nature and outcome of febrile episodes in patients with pancreatic and hepatobiliary cancer. Support. Care Cancer.

[87] Xu C., Lv P.-H., Huang X.-E., Wang S.-X., Sun L., Wang F.-A. (2014). Analysis of different ways of drainage for obstructive jaundice caused by hilar cholangiocarcinoma. Asian Pac. J. Cancer Prev..

[88] Aljahdli E.S. (2018). Management of distal malignant biliary obstruction. Saudi J. Gastroenterol..

[89] Avritscher E.B.C., Cooksley C.D., Rolston K.V., Swint J.M., Delclos G.L., Franzini L., Swisher S.G., Walsh G.L., Mansfield P.F., Elting L.S. (2014). Serious postoperative infections following resection of common solid tumors: Outcomes, costs, and impact of hospital surgical volume. Support. Care Cancer.

[90] Yang K., Zang Z.-Y., Niu K.-F., Sun L.-F., Zhang W.-H., Zhang Y.-X., Chen X.-L., Zhou Z.-G., Hu J.-K. (2021). The survival benefit and safety of splenectomy for gastric cancer with total gastrectomy: Updated results. Front. Oncol..

[91] Lee S.S., Morgenstern L., Phillips E.H., Hiatt J.R., Margulies D.R. (2000). Splenectomy for splenic metastases: A changing clinical spectrum. Am. Surg..

[92] Feola A., Niola M., Conti A., Delbon P., Graziano V., Paternoster M., Della Pietra B. (2016). Iatrogenic splenic injury: Review of the literature and medico-legal issues. Open Med..

[93] Di Sabatino A., Carsetti R., Corazza G.R. (2011). Post-Splenectomy and hyposplenic states. Lancet.

[94] Buzelé R., Barbier L., Sauvanet A., Fantin B. (2016). Medical complications following splenectomy. J. Visc. Surg..

[95] Pawelec G. (1999). Immunosenescence: Impact in the young as well as the old?. Mech. Ageing Dev..

[96] Eşme M., Topeli A., Yavuz B.B.D., Akova M. (2019). Infections in the elderly Critically-III patients. Front. Med..

[97] Tannou T., Koeberle S., Manckoundia P., Aubry R. (2019). Multifactorial immunodeficiency in frail elderly patients: Contributing factors and management. Med. Mal. Infect..

[98] Lyman G.H., Abella E., Pettengell R. (2014). Risk factors for febrile neutropenia among patients with cancer receiving chemotherapy: A systematic review. Crit. Rev. Oncol. Hematol..

[99] Balducci L., Hardy C.L., Lyman G.H. (2005). Hemopoiesis and aging. Cancer Treat. Res..

[100] Gay L., Melenotte C., Lakbar I., Mezouar S., Devaux C., Raoult D., Bendiane M.-K., Leone M., Mège J.-L. (2021). Sexual dimorphism and gender in infectious diseases. Front. Immunol..

[101] García-Gómez E., González-Pedrajo B., Camacho-Arroyo I. (2013). Role of Sex Steroid Hormones in Bacterial-Host Interactions. BioMed Res. Int..

[102] Ahmed S.A., Karpuzoglu E., Khan D., Klein S.L., Roberts C.W. (2010). Effects of sex steroids on innate and adaptive immunity. Sex Hormones and Immunity to Infection.

[103] Fish E.N. (2008). The X-files in immunity: Sex-Based differences predispose immune responses. Nat. Rev. Immunol..

[104] Harrington R.D., Hooton T.M. (2000). Urinary tract infection risk factors and gender. J. Gend. Specif. Med..

[105] Abdel-Rahman O. (2019). Impact of sex on chemotherapy toxicity and efficacy among patients with metastatic colorectal cancer: Pooled analysis of 5 randomized trials. Clin. Color. Cancer.

[106] Fontanella C., Bolzonello S., Lederer B., Aprile G. (2014). Management of breast cancer patients with chemotherapy-induced neutropenia or febrile neutropenia. Breast Care.

[107] Özdemir B.C., Csajka C., Dotto G.-P., Wagner A.D. (2018). Sex differences in efficacy and toxicity of systemic treatments: An undervalued issue in the era of precision oncology. J. Clin. Oncol..

[108] Ruzzo A., Graziano F., Galli F., Galli F., Rulli E., Lonardi S., Ronzoni M., Massidda B., Zagonel V., Pella N. (2019). Sex-Related Differences in impact on safety of pharmacogenetic profile for colon cancer patients treated with FOLFOX-4 or XELOX adjuvant chemotherapy. Sci. Rep..

[109] Bossi P., Delrio P., Mascheroni A., Zanetti M. (2021). The spectrum of malnutrition/cachexia/sarcopenia in oncology according to different cancer types and settings: A narrative review. Nutrients.

[110] Chandra R.K. (1996). Nutrition, immunity and infection: From basic knowledge of dietary manipulation of immune responses to practical application of ameliorating suffering and improving survival. Proc. Natl. Acad. Sci. USA.

[111] Triarico S., Rinninella E., Cintoni M., Capozza M.A., Mastrangelo S., Mele M.C., Ruggiero A. (2019). Impact of malnutrition on survival and infections among pediatric patients with cancer: A retrospective study. Eur. Rev. Med. Pharmacol. Sci..

[112] Falagas M.E., Kompoti M. (2006). Obesity and infection. Lancet Infect. Dis..

[113] Ghilotti F., Bellocco R., Ye W., Adami H.-O., Lagerros Y.T. (2019). Obesity and risk of infections: Results from men and women in the Swedish National March Cohort. Int. J. Epidemiol..

[114] Huttunen R., Syrjänen J. (2013). Obesity and the risk and outcome of infection. Int. J. Obes..

[115] Carey I.M., Critchley J.A., DeWilde S., Harris T., Hosking F.J., Cook D.G. (2018). Risk of infection in type 1 and type 2 diabetes compared with the general population: A matched cohort study. Diabetes Care.

[116] Berman S.J., Johnson E.W., Nakatsu C., Alkan M., Chen R., LeDuc J. (2004). Burden of infection in patients with end-stage renal disease requiring long-term dialysis. Clin. Infect. Dis..

[117] Cohen G., Hörl W.H. (2012). Immune dysfunction in Uremia—An update. Toxins.

[118] Lange P. (2009). Chronic obstructive pulmonary disease and risk of infection. Pneumonol. Alergol. Polska.

[119] Fragoulis G.E., Sipsas N.V. (2019). When rheumatology and infectious disease come together. Ther. Adv. Musculoskelet. Dis..

[120] Hsu C.-Y., Ko C.-H., Wang J.-L., Hsu T.-C., Lin C.-Y. (2019). Comparing the burdens of opportunistic infections among patients with systemic rheumatic diseases: A nationally representative cohort study. Arthritis Res..

[121] McDonagh T.A., Metra M., Adamo M., Gardner R.S., Baumbach A., Böhm M., Burri H., Butler J., Čelutkienė J., Chioncel O. (2021). 2021 ESC Guidelines for the diagnosis and treatment of acute and chronic heart failure: Developed by the task force for the diagnosis and treatment of acute and chronic heart failure of the European Society of Cardiology (ESC) with the special contribution of the Heart Failure Association (HFA) of the ESC. Eur. Heart J..

[122] Fernández J., Gustot T. (2012). Management of bacterial infections in cirrhosis. J. Hepatol..

[123] McCusker C., Warrington R. (2011). Primary immunodeficiency. Allergy Asthma Clin. Immunol..

[124] Okishiro M., Kim S.J., Tsunashima R., Nakayama T., Shimazu K., Shimomura A., Maruyama N., Tamaki Y., Noguchi S. (2012). MDM2 SNP309 and TP53 R72P associated with severe and febrile neutropenia in breast cancer patients treated with 5-FU/epirubicin/cyclophosphamide. Breast Cancer Res. Treat..

[125] Vulsteke C., Lambrechts D., Dieudonné A., Hatse S., Brouwers B., van Brussel T., Neven P., Belmans A., Schöffski P., Paridaens R. (2013). Genetic variability in the multidrug resistance associated protein-1 (ABCC1/MRP1) predicts hematological toxicity in breast cancer patients receiving (neo-)adjuvant chemotherapy with 5-fluorouracil, epirubicin and cyclophosphamide (FEC). Ann. Oncol..

[126] McLeod H.L., Sargent D., Marsh S., Green E.M., King C.R., Fuchs C.S., Ramanathan R.K., Williamson S.K., Findlay B.P., Thibodeau S.N. (2010). Pharmacogenetic predictors of adverse events and response to chemotherapy in metastatic colorectal cancer: Results from north american gastrointestinal intergroup trial N9741. J. Clin. Oncol..

[127] Cremolini C., Del Re M., Antoniotti C., Lonardi S., Bergamo F., Loupakis F., Borelli B., Marmorino F., Citi V., Cortesi E. (2017). DPYD and UGT1A1 genotyping to predict adverse events during first-line FOLFIRI or FOLFOXIRI plus bevacizumab in metastatic colorectal cancer. Oncotarget.

[128] Yamaguchi T., Iwasa S., Shoji H., Honma Y., Takashima A., Kato K., Hamaguchi T., Higuchi K., Boku N. (2019). Association between UGT1A1 gene polymorphism and safety and efficacy of irinotecan monotherapy as the third-line treatment for advanced gastric cancer. Gastric Cancer.

[129] Wood A.J., Pizzo P.A. (1993). Management of fever in patients with cancer and treatment-induced neutropenia. N. Engl. J. Med..

[130] DiNubile M.J. (1995). Fever and neutropenia: Still a challenge. Contemp. Intern. Med..

[131] Zell J.A., Chang J.C. (2005). Neoplastic fever: A neglected paraneoplastic syndrome. Support. Care Cancer.

[132] Kasuga I., Makino S., Kiyokawa H., Katoh H., Ebihara Y., Ohyashiki K. (2001). Tumor-Related leukocytosis is linked with poor prognosis in patients with lung carcinoma. Cancer.

[133] Hart P.C., Rajab I.M., Alebraheem M., Potempa L.A. (2020). C-Reactive protein and cancer—Diagnostic and therapeutic insights. Front. Immunol..

[134] Vincenzi B., Fioroni I., Pantano F., Angeletti S., Dicuonzo G., Zoccoli A., Santini D., Tonini G. (2016). Procalcitonin as diagnostic marker of infection in solid tumors patients with fever. Sci. Rep..

[135] Palmore T.N., Parta M., Cuellar-Rodriguez J., Gea-Banacloche J.C., Vincent T.D., Theodore S.L., Steven A.R. (2011). Infections in the Cancer Patient. DeVita, Hellman, and Rosenberg’s Cancer: Principles & Practice of Oncology.

[136] Gao Y., Shang Q., Li W., Guo W., Stojadinovic A., Mannion C., Man Y.-G., Chen T. (2020). Antibiotics for cancer treatment: A double-edged sword. J. Cancer.

[137] Hecker M.T., Aron D.C., Patel N.P., Lehmann M.K., Donskey C.J. (2003). Unnecessary use of antimicrobials in hospitalized patients: Current patterns of misuse with an emphasis on the antianaerobic spectrum of activity. Arch. Intern. Med..

[138] Fridkin S., Baggs J., Fagan R., Magill S., Pollack L.A., Malpiedi P., Slayton R., Khader K., Rubin M.A., Jones M. (2014). Vital signs: Improving antibiotic use among hospitalized patients. MMWR. Morb. Mortal. Wkly. Rep..

[139] Dellit T.H., Owens R.C., McGowan J.E., Gerding D.N., Weinstein R.A., Burke J.P., Huskins W.C., Paterson D.L., Fishman N.O., Carpenter C.F. (2007). Infectious diseases society of america and the society for healthcare epidemiology of america guidelines for developing an institutional program to enhance antimicrobial stewardship. Clin. Infect. Dis..

[140] Islas-Muñoz B., Volkow-Fernández P., Ibanes-Gutiérrez C., Villamar-Ramírez A., Vilar-Compte D., Cornejo-Juárez P. (2018). Bloodstream infections in cancer patients. Risk factors associated with mortality. Int. J. Infect. Dis..

[141] Baur D., Gladstone B.P., Burkert F., Carrara E., Foschi F., Döbele S., Tacconelli E. (2017). Effect of antibiotic stewardship on the incidence of infection and colonisation with antibiotic-resistant bacteria and Clostridium difficile infection: A systematic review and meta-analysis. Lancet Infect. Dis..

[142] Nathwani D., Varghese D., Stephens J., Ansari W., Martin S., Charbonneau C. (2019). Value of hospital antimicrobial stewardship programs [ASPs]: A systematic review. Antimicrob. Resist. Infect. Control..

[143] Ramos-Casals M., Brahmer J.R., Callahan M.K., Flores-Chávez A., Keegan N., Khamashta M.A., Lambotte O., Mariette X., Prat A., Suárez-Almazor M.E. (2020). Immune-Related adverse events of checkpoint inhibitors. Nat. Rev. Dis. Prim..

[144] Del Castillo M., Romero F.A., Argüello E., Kyi C., Postow M.A., Redelman-Sidi G. (2016). The spectrum of serious infections among patients receiving immune checkpoint blockade for the treatment of melanoma. Clin. Infect. Dis..

[145] Elkrief A., DeRosa L., Kroemer G., Zitvogel L., Routy B. (2019). The negative impact of antibiotics on outcomes in cancer patients treated with immunotherapy: A new independent prognostic factor?. Ann. Oncol..

[146] Freifeld A.G., Bow E.J., Sepkowitz K.A., Boeckh M.J., Ito J.I., Mullen C.A., Raad I.I., Rolston K.V., Young J.-A.H., Wingard J.R. (2011). Clinical practice guideline for the use of antimicrobial agents in neutropenic patients with cancer: 2010 update by the infectious diseases society of america. Clin. Infect. Dis..

[147] Carmona-Bayonas A., Jiménez-Fonseca P., Echaburu J.V., Cánovas M.S., De La Peña F.A. (2017). The time has come for new models in febrile neutropenia: A practical demonstration of the inadequacy of the MASCC score. Clin. Transl. Oncol..

[148] Peyrony O., Gerlier C., Barla I., Ellouze S., Legay L., Azoulay E., Chevret S., Fontaine J.-P. (2020). Antibiotic prescribing and outcomes in cancer patients with febrile neutropenia in the emergency department. PLoS ONE.

[149] Elting L.S., Lu C., Escalante C.P., Giordano S.H., Trent J.C., Cooksley C., Avritscher E.B., Shih Y.-C.T., Ensor J., Bekele B.N. (2008). Outcomes and cost of outpatient or inpatient management of 712 patients with febrile neutropenia. J. Clin. Oncol..

[150] AJMC Guidelines in the Management of Febrile Neutropenia for Clinical Practice. https://www.ajmc.com/view/guidelines-in-the-management-of-febrile-neutropenia-for-clinical-practice.

[151] Taplitz R.A., Kennedy E.B., Bow E.J., Crews J., Gleason C., Hawley D.K., Langston A.A., Nastoupil L.J., Rajotte M., Rolston K. (2018). Outpatient management of fever and neutropenia in adults treated for malignancy: American society of clinical oncology and infectious diseases society of america clinical practice guideline update. J. Clin. Oncol..

[152] Anatoliotaki M., Valatas V., Mantadakis E., Apostolakou H., Mavroudis D., Georgoulias V., Rolston K.V., Kontoyiannis D.P., Galanakis E., Samonis G. (2004). Bloodstream infections in patients with solid tumors: Associated factors, microbial spectrum and outcome. Infection.

[153] Marín M., Gudiol C., Garcia-Vidal C., Ardanuy C., Carratala J. (2014). Bloodstream Infections in patients with solid tumors: Epidemiology, antibiotic therapy, and outcomes in 528 episodes in a single cancer center. Medicine.

[154] Seifert H., Cornely O., Seggewiss K., Decker M., Stefanik D., Wisplinghoff H., Fätkenheuer G. (2003). Bloodstream infection in neutropenic cancer patients related to short-term nontunnelled catheters determined by quantitative blood cultures, differential time to positivity, and molecular epidemiological typing with pulsed-field gel electrophoresis. J. Clin. Microbiol..

[155] Mermel L.A., Allon M., Bouza E., Craven D.E., Flynn P., O’Grady N.P., Raad I.I., Rijnders B.J.A., Sherertz R.J., Warren D.K. (2009). Clinical practice guidelines for the diagnosis and management of intravascular catheter-related infection: 2009 update by the infectious diseases society of america. Clin. Infect. Dis..

[156] Cantón-Bulnes M.L., Garnacho-Montero J. (2019). Practical approach to the management of catheter-related bloodstream infection. Rev. Esp. Quimioter.

[157] Muff S., Tabah A., Que Y.-A., Timsit J.-F., Mermel L., Harbarth S., Buetti N. (2021). Short-Course versus long-course systemic antibiotic treatment for uncomplicated intravascular catheter-related bloodstream infections due to gram-negative bacteria, enterococci or coagulase-negative staphylococci: A systematic review. Infect. Dis. Ther..

[158] Chaves F., Garnacho-Montero J., Del Pozo J.L., Bouza E., Capdevila J., de Cueto M., Domínguez M., Esteban J., Fernández-Hidalgo N., Sampedro M.F. (2018). Diagnosis and treatment of catheter-related bloodstream infection: Clinical guidelines of the Spanish Society of Infectious Diseases and Clinical Microbiology and (SEIMC) and the Spanish Society of Spanish Society of Intensive and Critical Care Medicine and Coronary Units (SEMICYUC). Med. Intensiv..

[159] Norris L.B., Kablaoui F., Brilhart M.K., Bookstaver P.B. (2017). Systematic review of antimicrobial lock therapy for prevention of central-line-associated bloodstream infections in adult and pediatric cancer patients. Int. J. Antimicrob. Agents.

[160] Pliakos E.E., Andreatos N., Ziakas P., Mylonakis E. (2019). The cost-effectiveness of antimicrobial lock solutions for the prevention of central line–associated bloodstream infections. Clin. Infect. Dis..

[161] Robinson J.L., Tawfik G., Saxinger L., Stang L., Etches W., Lee B. (2005). Stability of heparin and physical compatibility of heparin/antibiotic solutions in concentrations appropriate for antibiotic lock therapy. J. Antimicrob. Chemother..

[162] Luther M.K., Mermel L.A., Laplante K.L. (2017). Comparison of linezolid and vancomycin lock solutions with and without heparin against biofilm-producing bacteria. Am. J. Health Pharm..

[163] Del Pozo J.L. (2009). Role of antibiotic lock therapy for the treatment of catheter-related bloodstream infections. Int. J. Artif. Organs.

[164] Bookstaver P.B., Rokas K.E.E., Norris L.B., Edwards J.M., Sherertz R.J. (2013). Stability and compatibility of antimicrobial lock solutions. Am. J. Health Syst. Pharm..

[165] LaPlante K.L., Mermel L.A. (2007). In Vitro activity of daptomycin and vancomycin lock solutions on staphylococcal biofilms in a central venous catheter model. Nephrol. Dial. Transpl..

[166] Krishnasami Z., Carlton D., Bimbo L., Taylor M.E., Balkovetz D.F., Barker J., Allon M. (2002). Management of hemodialysis catheter-related bacteremia with an adjunctive antibiotic lock solution. Kidney Int..

[167] Vercaigne L.M., Sitar D.S., Penner S.B., Bernstein K., Wang G.Q., Burczynski F. (2000). Antibiotic-Heparin lock: In Vitro antibiotic stability combined with heparin in a central venous catheter. Pharmacotherapy.

[168] Justo J.A., Bookstaver P.B. (2014). Antibiotic lock therapy: Review of technique and logistical challenges. Infect. Drug Resist..

[169] Rijnders B.J., Van Wijngaerden E., Vandecasteele S.J., Stas M., Peetermans W.E. (2005). Treatment of long-term intravascular catheter-related bacteraemia with antibiotic lock: Randomized, placebo-controlled trial. J. Antimicrob. Chemother..

[170] Lee J.-Y., Ko K.S., Peck K.R., Oh W.S., Song J.-H. (2006). In Vitro evaluation of the antibiotic lock technique (ALT) for the treatment of catheter-related infections caused by staphylococci. J. Antimicrob. Chemother..

[171] Droste J.C., Jeraj H.A., Macdonald A., Farrington K. (2003). Stability and in vitro efficacy of antibiotic-heparin lock solutions potentially useful for treatment of central venous catheter-related sepsis. J. Antimicrob. Chemother..

[172] Lee M.Y., Ko K.S., Song J.-H., Peck K.R. (2007). In Vitro effectiveness of the antibiotic lock technique (ALT) for the treatment of catheter-related infections by Pseudomonas aeruginosa and Klebsiella pneumoniae. J. Antimicrob. Chemother..

[173] Onland W., Shin C.E., Fustar S., Rushing T., Wong W.-Y. (2006). Ethanol-Lock technique for persistent bacteremia of long-term intravascular devices in pediatric patients. Arch. Pediatr. Adolesc. Med..

[174] EMC Ampicillin 500 mg powder for solution for injection—Summary of Product Characteristics (SPC). https://www.medicines.org.uk/emc/product/12892/smpc#gref.

[175] Vila-Corcoles A., Ochoa-Gondar O., Rodriguez-Blanco T., Raga-Luria X., Gomez-Bertomeu F. (2009). Epidemiology of community-acquired pneumonia in older adults: A population-based study. Respir. Med..

[176] Parakh A., Krishnamurthy S., Bhattacharya M. (2009). Ertapenem. Kathmandu Univ. Med. J. (KUMJ).

[177] Kalil A.C., Metersky M.L., Klompas M., Muscedere J., Sweeney D.A., Palmer L.B., Napolitano L.M., O’Grady N.P., Bartlett J.G., Carratala J. (2016). Management of adults with hospital-acquired and ventilator-associated pneumonia: 2016 clinical practice guidelines by the infectious diseases society of america and the american thoracic society. Clin. Infect. Dis..

[178] Metlay J.P., Waterer G.W., Long A.C., Anzueto A., Brozek J., Crothers K., Cooley L.A., Dean N.C., Fine M.J., Flanders S.A. (2019). Diagnosis and treatment of adults with community-acquired pneumonia. An official clinical practice guideline of the american thoracic society and infectious diseases society of America. Am. J. Respir. Crit. Care Med..

[179] Hanson K.E., Azar M.M., Banerjee R., Chou A., Colgrove R.C., Ginocchio C.C., Hayden M.K., Holodiny M., Jain S., Koo S. (2020). Molecular testing for acute respiratory tract infections: Clinical and diagnostic recommendations from the IDSA’s diagnostics committee. Clin. Infect. Dis..

[180] Sartelli M., Chichom-Mefire A., Labricciosa F.M., Hardcastle T., Abu-Zidan F.M., Adesunkanmi A.K., Ansaloni L., Bala M., Balogh Z.J., Beltrán M.A. (2017). The management of intra-abdominal infections from a global perspective: 2017 WSES guidelines for management of intra-abdominal infections. World J. Emerg. Surg..

[181] Solomkin J.S., Mazuski J.E., Bradley J.S., Rodvold K.A., Goldstein E.J., Baron E.J., O’Neill P.J., Chow A.W., Dellinger E.P., Eachempati S.R. (2010). Diagnosis and management of complicated intra-abdominal infection in adults and children: Guidelines by the surgical infection society and the infectious diseases society of america. Clin. Infect. Dis..

[182] Guevara E.A.Y., Aitken S.L., Olvera A.V., Carlin L., Fernandes K.E., Bhatti M.M., Garey K.W., Adachi J., Okhuysen P.C. (2020). Clostridioides difficile infection in cancer and immunocompromised patients: Relevance of a two-step diagnostic algorithm and infecting ribotypes on clinical outcomes. Clin. Infect. Dis..

[183] Abughanimeh O., Qasrawi A., Kaddourah O., Al Momani L., Abu Ghanimeh M. (2018). Clostridium difficileinfection in oncology patients: Epidemiology, pathophysiology, risk factors, diagnosis, and treatment. Hosp. Pract..

[184] Johnson S., Lavergne V., Skinner A.M., Gonzales-Luna A.J., Garey K.W., Kelly C.P., Wilcox M.H. (2021). Clinical practice guideline by the infectious diseases society of america (idsa) and society for Healthcare Epidemiology of America (SHEA): 2021 focused update guidelines on management of clostridioides difficile infection in adults. Clin. Infect. Dis..

[185] Kirkpatrick I.D.C., Greenberg H.M. (2003). Gastrointestinal complications in the neutropenic patient: Characterization and differentiation with abdominal CT. Radiology.

[186] Song H.K., Kreisel D., Canter R., Krupnick A.S., Stadtmauer E.A., Buzby G. (1998). Changing presentation and management of neutropenic enterocolitis. Arch. Surg..

[187] Tigabu A., Ferede W., Belay G., Gelaw B. (2020). Prevalence of asymptomatic bacteriuria and antibiotic susceptibility patterns of bacterial isolates among cancer patients and healthy blood donors at the University of Gondar Specialized Hospital. Int. J. Microbiol..

[188] Shrestha G., Wei X., Hann K., Soe K., Satyanarayana S., Siwakoti B., Bastakoti S., Mulmi R., Rana K., Lamichhane N. (2021). Bacterial profile and antibiotic resistance among cancer patients with urinary tract infection in a national tertiary cancer hospital of Nepal. Trop. Med. Infect. Dis..

[189] Parikh P., Bhat V. (2015). Urinary tract infection in cancer patients in a tertiary cancer setting in India: Microbial spectrum and antibiotic susceptibility pattern. Antimicrob. Resist. Infect. Control.

[190] Khaparkuntikar M., Siddiqui N., Bhirud P. (2017). Urinary tract infection in cancer patients at Government Cancer Hospital Aurangabad, India. Int. J. Curr. Microbiol. Appl. Sci..

[191] Bonkat G., Bartoletti R., Bruyère F., Cai T., Geerlings S.E., Köves B., Schubert S., Wagenlehner F. (2020). EAU Guidelines on Urological Infections.

[192] Nicolle L. (2005). Complicated urinary tract infection in adults. Can. J. Infect. Dis. Med. Microbiol..

[193] Lopes M.S.M., Machado L.M., Silva P.A.I.A., Uchiyama A.A.T., Yen C.T., Ricardo E.D., Mutao T.S., Pimenta J.R., Shimba D.S., Hanriot R.M. (2020). Antibiotics, cancer risk and oncologic treatment efficacy: A practical review of the literature. Ecancermedicalscience.

[194] Shui L., Yang X., Li J., Yi C., Sun Q., Zhu H. (2020). Gut microbiome as a potential factor for modulating resistance to cancer immunotherapy. Front. Immunol..

[195] Ma W., Mao Q., Xia W., Dong G., Yu C., Jiang F. (2019). Gut microbiota shapes the efficiency of cancer therapy. Front. Microbiol..

[196] Reed J.P., Devkota S., Figlin R.A. (2019). Gut microbiome, antibiotic use, and immunotherapy responsiveness in cancer. Ann. Transl. Med..

[197] Gopalakrishnan V., Spencer C.N., Nezi L., Reuben A., Andrews M.C., Karpinets T.V., Prieto P.A., Vicente D., Hoffman K., Wei S.C. (2018). Gut microbiome modulates response to anti–PD-1 immunotherapy in melanoma patients. Science.

[198] Matson V., Fessler J., Bao R., Chongsuwat T., Zha Y., Alegre M.-L., Luke J.J., Gajewski T.F. (2018). The commensal microbiome is associated with anti–PD-1 efficacy in metastatic melanoma patients. Science.

[199] Routy B., Le Chatelier E., DeRosa L., Duong C.P.M., Alou M.T., Daillère R., Fluckiger A., Messaoudene M., Rauber C., Roberti M.P. (2018). Gut microbiome influences efficacy of PD-1–based immunotherapy against epithelial tumors. Science.

[200] Vétizou M., Pitt J.M., Daillère R., Lepage P., Waldschmitt N., Flament C., Rusakiewicz S., Routy B., Roberti M.P., Duong C.P.M. (2015). Anticancer immunotherapy by CTLA-4 blockade relies on the gut microbiota. Science.

[201] Sivan A., Corrales L., Hubert N., Williams J.B., Aquino-Michaels K., Earley Z.M., Benyamin F.W., Lei Y.M., Jabri B., Alegre M.-L. (2015). Commensal Bifidobacterium promotes antitumor immunity and facilitates anti-PD-L1 efficacy. Science.

[202] Chaput N., Lepage P., Coutzac C., Soularue E., Le Roux K., Monot C., Boselli L., Routier E., Cassard L., Collins M. (2017). Baseline gut microbiota predicts clinical response and colitis in metastatic melanoma patients treated with ipilimumab. Ann. Oncol..

[203] Tinsley N., Zhou C., Tan G., Rack S., Lorigan P., Blackhall F., Krebs M., Carter L., Thistlethwaite F., Graham D. (2020). Cumulative antibiotic use significantly decreases efficacy of checkpoint inhibitors in patients with advanced cancer. Oncologist.

[204] Mohiuddin J.J., Chu B., Facciabene A., Poirier K., Wang X., Doucette A., Zheng C., Xu W., Anstadt E.J., Amaravadi R.K. (2020). Association of antibiotic exposure with survival and toxicity in patients with melanoma receiving immunotherapy. J. Natl. Cancer Inst..

[205] Derosa L., Hellmann M., Spaziano M., Halpenny D., Fidelle M., Rizvi H., Long N., Plodkowski A., Arbour K., Chaft J. (2018). Negative association of antibiotics on clinical activity of immune checkpoint inhibitors in patients with advanced renal cell and non-small-cell lung cancer. Ann. Oncol..

[206] Rubio X.M., Chara L., Sotelo-Lezama M., Castro R.L., Rubio-Martínez J., Velastegui A., Olier-Garate C., Falagan S., Gómez-Barreda I., Bautista-Sanz P. (2018). MA10.01 antibiotic use and PD-1 inhibitors: Shorter survival in lung cancer, especially when given intravenously. Type of infection also matters. J. Thorac. Oncol..

[207] Galli G., Triulzi T., Proto C., Signorelli D., Imbimbo M., Poggi M., Fucà G., Ganzinelli M., Vitali M., Palmieri D. (2019). Association between antibiotic-immunotherapy exposure ratio and outcome in metastatic non small cell lung cancer. Lung Cancer.

[208] Geum M., Kim C., Kang J., Choi J., Kim J., Son E., Lim S., Rhie S. (2021). Broad-Spectrum antibiotic regimen affects survival in patients receiving nivolumab for non-small cell lung cancer. Pharmaceuticals.

[209] Lalani A.-K.A., Xie W., Braun D.A., Kaymakcalan M., Bossé D., Steinharter J.A., Martini D., Simantov R., Lin X., Wei X.X. (2020). Effect of antibiotic use on outcomes with systemic therapies in metastatic renal cell carcinoma. Eur. Urol. Oncol..

[210] Huang X.-Z., Gao P., Song Y.-X., Xu Y., Sun J.-X., Chen X.-W., Zhao J.-H., Wang Z.-N. (2019). Antibiotic use and the efficacy of immune checkpoint inhibitors in cancer patients: A pooled analysis of 2740 cancer patients. OncoImmunology.

[211] Lurienne L., Cervesi J., Duhalde L., de Gunzburg J., Andremont A., Zalcman G., Buffet R., Bandinelli P.-A. (2020). NSCLC immunotherapy efficacy and antibiotic use: A systematic review and meta-analysis. J. Thorac. Oncol..

[212] Wilson B.E., Routy B., Nagrial A., Chin V.T. (2020). The effect of antibiotics on clinical outcomes in immune-checkpoint blockade: A systematic review and meta-analysis of observational studies. Cancer Immunol. Immunother..

[213] Uribe-Herranz M., Rafail S., Beghi S., Gil-De-Gómez L., Verginadis I., Bittinger K., Pustylnikov S., Pierini S., Perales-Linares R., Blair I.A. (2020). Gut microbiota modulate dendritic cell antigen presentation and radiotherapy-induced antitumor immune response. J. Clin. Investig..

[214] Yang K., Hou Y., Zhang Y., Liang H., Sharma A., Zheng W., Wang L., Torres R., Tatebe K., Chmura S.J. (2021). Suppression of local type I interferon by gut microbiota–derived butyrate impairs antitumor effects of ionizing radiation. J. Exp. Med..

[215] Nenclares P., Bhide S.A., Sandoval-Insausti H., Pialat P., Gunn L., Melcher A., Newbold K., Nutting C.M., Harrington K.J. (2020). Impact of antibiotic use during curative treatment of locally advanced head and neck cancers with chemotherapy and radiotherapy. Eur. J. Cancer.

[216] Corty R.W., Langworthy B.W., Fine J.P., Buse J.B., Sanoff H.K., Lund J.L. (2020). Antibacterial Use Is Associated with an Increased Risk of Hematologic and Gastrointestinal Adverse Events in Patients Treated with Gemcitabine for Stage IV Pancreatic Cancer. Oncologist.

[217] Lee N., Kim W.-U. (2017). Microbiota in T-cell homeostasis and inflammatory diseases. Exp. Mol. Med..

